# A novel role for NUPR1 in the keratinocyte stress response to UV oxidized phospholipids

**DOI:** 10.1016/j.redox.2018.11.006

**Published:** 2018-11-13

**Authors:** Marie-Sophie Narzt, Ionela-Mariana Nagelreiter, Olga Oskolkova, Valery N. Bochkov, Julie Latreille, Maria Fedorova, Zhixu Ni, Fernando J. Sialana, Gert Lubec, Manuel Filzwieser, Maria Laggner, Martin Bilban, Michael Mildner, Erwin Tschachler, Johannes Grillari, Florian Gruber

**Affiliations:** a Department of Dermatology, Medical University of Vienna, Vienna, Austria; b Christian Doppler Laboratory for Biotechnology of Skin Aging, Austria; c Institute of Pharmaceutical Sciences, University of Graz, Graz, Austria; d Department of Biology & Women's Beauty, Chanel, Pantin, France; e Institute of Bioanalytical Chemistry, Faculty of Chemistry, Universität Leipzig, Leipzig, Germany; f Center for Biotechnology and Biomedicine, Universität Leipzig, Leipzig, Germany; g Department of Pharmaceutical Chemistry, Faculty of Life Sciences, University of Vienna, Vienna, Austria; h Paracelsus Medical University of Salzburg, Salzburg, Austria; i Department of Ophthalmology and Optometry, Medical University of Vienna, Vienna, Austria; j Department of Laboratory Medicine & Core Facility Genomics, Medical University of Vienna, Vienna, Austria; k Department of Biotechnology, BOKU, University of Natural Resources and Life Sciences Vienna, Austria

## Abstract

Ultraviolet light is the dominant environmental oxidative skin stressor and a major skin aging factor. We studied which oxidized phospholipid (OxPL) mediators would be generated in primary human keratinocytes (KC) upon exposure to ultraviolet A light (UVA) and investigated the contribution of OxPL to UVA responses. Mass spectrometric analysis immediately or 24 h post UV stress revealed significant changes in abundance of 173 and 84 lipid species, respectively. We identified known and novel lipid species including known bioactive and also potentially reactive carbonyl containing species. We found indication for selective metabolism and degradation of selected reactive lipids. Exposure to both UVA and to in vitro UVA - oxidized phospholipids activated, on transcriptome and proteome level, NRF2/antioxidant response signaling, lipid metabolizing enzyme expression and unfolded protein response (UPR) signaling. We identified NUPR1 as an upstream regulator of UVA/OxPL transcriptional stress responses and found this protein to be expressed in the epidermis. Silencing of NUPR1 resulted in augmented expression of antioxidant and lipid detoxification genes and disturbed the cell cycle, making it a potential key factor in skin reactive oxygen species (ROS) responses intimately involved in aging and pathology.

## Graphical abstract

**Figure:**
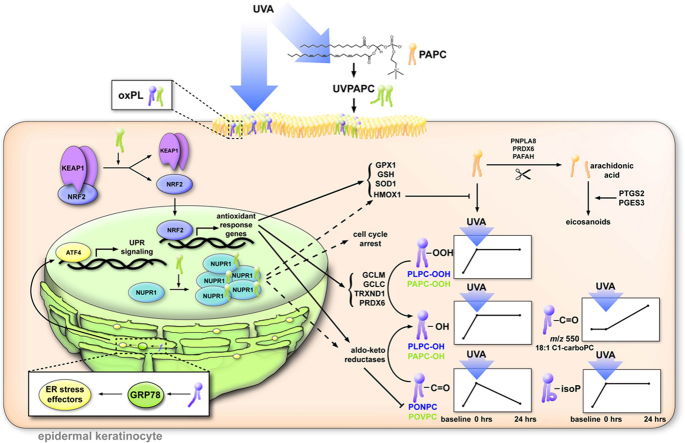

## 1 Introduction

The human skin is the organ most exposed to environmental oxidative assaults that cause cell damage, promote aging and result in pathologies. The dominant extrinsic oxidizing factor is ultraviolet A light (UVA, 340–400 nm) which can penetrate deeply into the skin and modifies nucleic acids, proteins and lipids [74]. The UVA induced DNA damage is mutagenic and promotes photoaging [4], the premature aging phenotype of excessively sun exposed skin [67]. Further, UVA causes oxidative modifications of proteins [57], rendering them dysfunctional and impairing their degradation [38]. Oxidized protein accumulates in photoaged skin [63] and promotes precancerous actinic elastosis [52] which is together with UV-induced constitutive matrix proteolysis a significant risk factor for keratinocyte- derived cancers of the skin [77].

Phospholipids containing (poly-) unsaturated fatty acid moieties which are present in all cellular membranes are prone to oxidation [59] and yield a wide array of UVA oxidation products [31]. Reactive oxidized lipid species modify DNA and proteins such as histones [20] thereby affecting cell signaling and epigenetics [26]. Bi-reactive lipid oxidation products like bis-aldehydes crosslink macromolecules [65] which can be detected in photo-aged skin [46], [79]. Signaling molecules like receptors are targets of lipid modification [37], contributing to the increasingly recognized effects of lipids on cellular signaling. Additionally to the chemically reactive lipids, potent lipid signaling molecules are formed by UV through enzymes [43] or non-enzymatically [34], [62]. Non-enzymatically oxidized 1-palmitoyl-2-arachidonoyl-*sn*-glycero-3-phosphocholine (PAPC) is an established model substance that contains bioactive lipids found in the circulation within oxidized low density lipoprotein (oxLDL) but also in the skin [64], [76]. Oxidation of PAPC yields phospholipid hydroperoxides, -hydroxides, isoprostanoids, endoperoxides, cyclopentenones, carbonyls and lysophosphatidylcholines, all identified by mass spectrometric (MS) methods [64], [71]. The individual lipids in these classes differ in their structure and chemical reactivity and, consequently in their biological activity.

Through agonism or antagonism of pattern recognition receptors, specific lipid classes can elicit quite distinct modulation of innate inflammation [68], [9]. In keratinocytes, phospholipid UV- oxidation products exert local immunosuppression as agonists of the platelet activating factor receptor [45]. As activators of nuclear factor erythroid 2 like 2 (NRF2), specific OxPL can exert additional immunomodulation [41], [61], and we have found UVA oxidized PL to be formed in cutaneous cells and to act via NRF2 [32], [33]. Further, OxPL initiate autophagy in KC, and genetic deletion of autophagy led to accumulation of crosslinked protein and of oxidized phospholipids [85].

Thus, to understand the contribution of UV-generated bioactive lipids to the impact of UV light on the skin, we need to identify the lipid species, their activity as signaling molecules and chemical modifiers, and how the cells further process the lipids or their adducts. In this study we investigated generation of OxPL in primary human epidermal keratinocytes through UVA, and their contribution to UV -regulation of the transcriptome and proteome of keratinocytes. Of the regulated OxPL we could assign twenty, and propose structures for five novel UV-regulated species. Investigating the transcriptome and the proteome of UVA- or UVPAPC- treated KC we identified NRF2 signaling, a UPR/ER stress signature and induction of lipid detoxifying genes as shared responses to both stressors. A bioinformatic analysis of upstream regulatory factors predicted nuclear protein 1 (NUPR1), to be involved in the stress regulation. NUPR1 is implicated in autophagy-, chromatin accessibility-, and transcriptional regulation in various tissues (rev. in [13]). We here report that expression of NUPR1 and downstream genes was induced by UVA and exposure to oxidized lipids. Knockdown of NUPR1 increased expression of HMOX1, the (lipid) detoxifying aldo-keto reductase AKR1C1 and impaired cell cycle progression. We localized NUPR1 in nuclei of epidermal keratinocytes and found, that exposure of recombinant NUPR1 to oxidized PAPC affected its electrophoretic mobility, potentially by modifying and crosslinking the protein.

Our data thus suggest a novel role for NUPR1 in the skin, as transcriptional regulator of redox responses, lipid metabolism and the cell cycle of epidermal keratinocytes under stress evoked by UV light and bioactive oxidized lipids.

## 2 Results

Throughout this study we compared the effects of UVA exposure on keratinocyte lipidome, transcriptome and proteome to the effects of externally addend UVA-oxidized phospholipids. While external addition of OxPL does not exactly model their intracellular generation, cells in an UV-exposed microenvironment are likely to encounter these very mediators, be it as highly amphipathic and membrane permeant lipid species [73] from vesicles, as “whiskers” - or danger associated molecular patterns (DAMPs) protruding from the membranes of cells or vesicles [30], as oxidation products on LDL particles [47], or among remnants of dead cells.

### 2.1 Experimental design

**Fig. 1:**
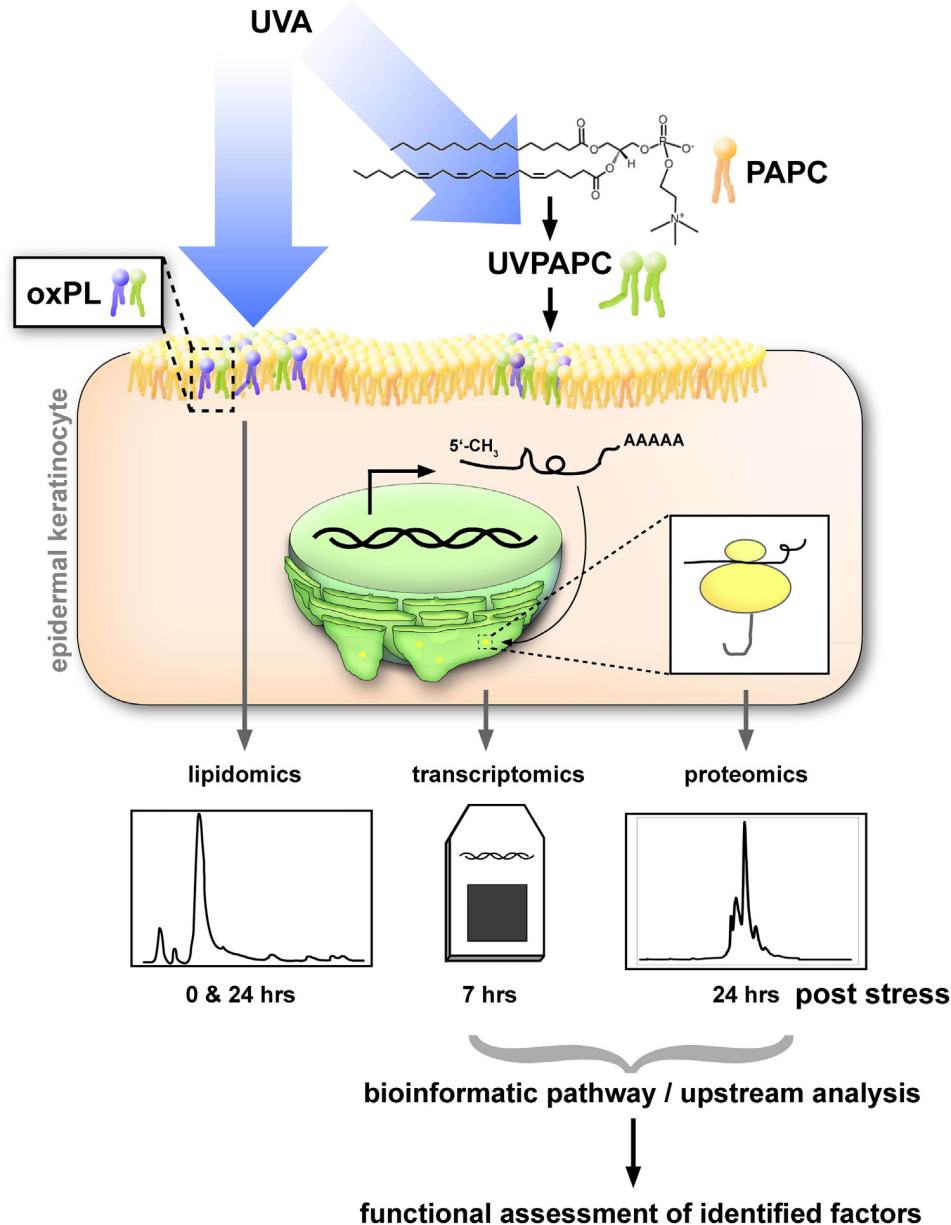
**Experimental design.** UVA was either applied directly to primary KC or to the polyunsaturated model phospholipid PAPC (yielding oxidized UVPAPC) which was then added to culture medium of KC. Immediately and twenty four hours after either treatment phospholipids were isolated from both cultures and sham treated controls and analyzed with HPLC-MS/MS. Seven hours post treatment, a transcriptomic analysis was performed, and twenty four hours after stress, proteomic analysis were performed with HPLC-MS/MS. The transcriptome and proteome datasets were analyzed for activation of signaling pathways and upstream regulatory factors. The newly identified regulatory factor NUPR1 was then functionally assessed by siRNA mediated knockdown in KC.

First, we investigated the effect of UVA-1 (340–400 nm, 40 J/cm^2^) exposure on phospholipid oxidation in primary human keratinocytes immediately after irradiation and after a twenty four hour recovery period. In parallel, we assessed the oxidized phospholipidome of cells to which 25 µg/ml externally photo-oxidized 1-palmitoyl-2-arachidonoyl-*sn*-glycero-3-phosphocholine (PAPC, UVPAPC when UV oxidized) had been added. We applied a semi- targeted lipidomic method using HPLC-electrospray ionization (ESI)-MS/MS that we have recently developed [31], and additionally a high-resolution MS method for structural identification of selected unknown PL species for these tasks. Further, using microarrays for transcriptomic profiling we assessed the effect of UVA- and UVPAPC treatment on KC seven hours post exposure, a timepoint at which we had previously studied UV- and UVPAPC mediated gene regulation in dermal fibroblasts [32]. We hypothesized that similar to what we observed in FB also in keratinocytes UVA- oxidized phospholipids would account for a part of the transcriptional UVA response. Additionally, we investigated the effect of both treatments on the proteome at twenty four hours post exposure using a LC-MS method. Using the data from transcriptomics and proteomics we performed an analysis on activation of signaling pathways and upstream regulators to predict factors responsible for the OxPL's contribution to UVA effects on mRNA and protein composition of KC. Finally, we used siRNA silencing to investigate the role of newly identified upstream regulators in KC and their UV response (Fig. 1).

### 2.2 Lipidomic analysis of UVA and UVPAPC exposed keratinocytes

We followed the changes in the relative abundance of the most prominent known and also unidentified oxidation products derived from PAPC but also from 1-stearoyl-2-arachidonoyl-*sn*-glycero-3-phosphocholine (SAPC), 1-palmitoyl-2-linoleoyl-*sn*-glycero-3-phosphocholine (PLPC) and 1-stearoyl-2-linoleoyl-*sn*-glycero-3-phosphocholine (SLPC) at 0 h and 24 h post UVA and UVPAPC exposure, respectively.

**Fig. 2:**
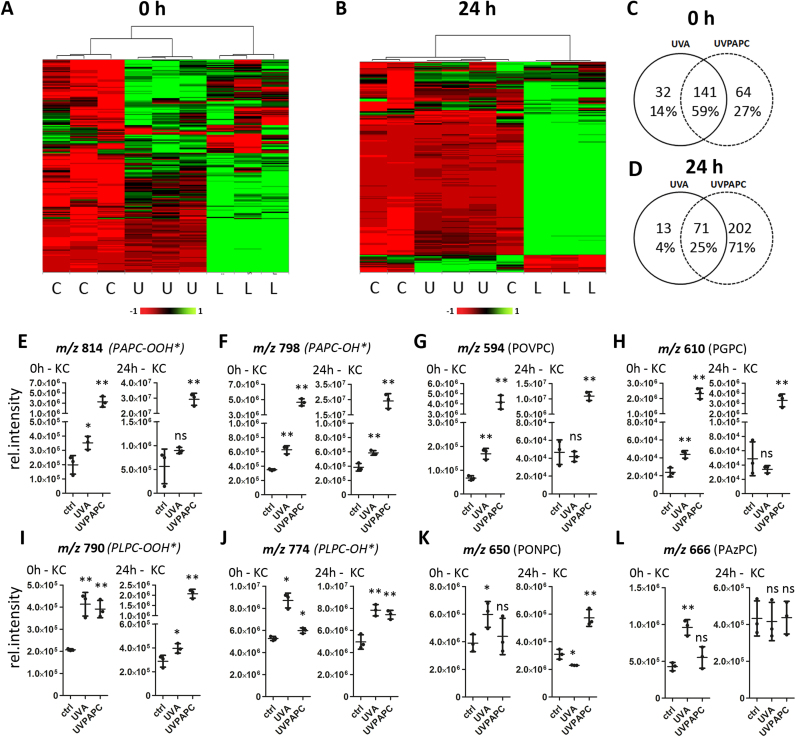
**Lipidomic analysis of UVA and UVPAPC exposed keratinocytes.** Polar lipids were isolated from human primary keratinocytes immediately and after 24 h post stress treatment with UVA (40 J/cm^2^) and UVPAPC (25 µg/ml), respectively. Oxidized phosphatdiylcholine species were quantified with HPLC MS/MS. **A,B** Heat-map analysis of the oxidized phospholipidome at 0 h and 24 h, respectively. Columns represent experimental treatment conditions; rows show abundance of individual OxPC species upon treatment (green, high abundance; red, low abundance; column identifiers: “C” - ctrl, “U” - UVA, “L” – UVPAPC). **C,D** Venn diagrams show numbers of significantly regulated OxPC species 0 h (C) and 24 h(D) after treatment. **E-L,** Dot plots show abundance of the respective species 0 h and 24 h post stress treatment normalized to 1,2-dipalmitoyl-*sn*-glycero-3-phosphocholine (DPPC) as internal standard. (n = 3; error bars indicate SD). Asterisks indicate significant differences (*P < 0.05; ** P < 0.01) determined by Student's *t*-test.

Immediately after exposure to UVA-1 (40 J/cm^2^), 173 oxPC species were significantly increased as compared to sham treatment. Addition of UVPAPC (25 µg/ml) to the cells followed by immediate extraction resulted in up-regulation of 205 oxidized PC species of which 141 species were increased in both conditions (UVA and UVPAPC treatment; Fig. 2A, C). UVA and UVPAPC treatment decreased abundance of one and three oxPCs below detection limit, respectively. A principal component analysis (PCA) of the first two principal components demonstrates high reproducibility within the replicates, whereas the different treatments could be clearly separated within the two dimensions (Supplementary Fig. 1A). After a 24 h recovery phase, 84 OxPCs were up-regulated in the UVA treated group, whereas 273 oxidized lipids were up-regulated by UVPAPC treatment (Fig. 2B, D). Seventy one of the UVA induced OxPCs were also increased upon UVPAPC exposure after 24 h (2D). PCA analysis (Supplementary Fig. 1B) and the heatmap at 24 h (Fig. 2B) indicated a high similarity between the UVA- exposed samples and the controls, but both groups were clearly distinct from the UVPAPC treated group. From the 173 species that had been significantly induced by UVA at 0 h, 119 species (59%) returned to baseline level after 24 h, whereas from 205 species increased after UVPAPC addition only 8 (3%) returned to the baseline. Species that were exclusively elevated over control at 24 h post stress amounted to 30 in the UVA treated cells and to 76 in the UVPAPC treated cells (Supplementary Fig. 1C). Together, these results indicate that the oxidized phospholipidome of UVA irradiated KC largely returned to baseline 24 h after exposure to UVA, while a smaller number of distinct OxPC species were induced after 24 h.

The earliest products of phospholipid (per-) oxidation are PL hydroperoxides (PL-OOH), which also represent a gold standard for cellular redox stress [22]. Quantifying these and the less reactive PL hydroxides (PL-OH), which mostly derive from the reduction of the PL –OOH gives an indication whether cells recover from redox stress in a given time. We quantified PC- hydroperoxides and –hydroxides derived from PAPC and PLPC and found in irradiated KC an immediate rise in hydroperoxides (Fig. 2 E, I). Exposure to UVPAPC led to strong increase in PAPC-OOH (most of which can be attributed to its presence in UVPAPC itself [34]), but also PLPC-OOH was immediately increased to a level comparable to what was observed after UVA exposure, an indicator that the cellular oxidative stress level upon both treatments was comparable. After 24 h, PL-OOH levels remained elevated but did not augment further in the UVA exposed cells, whereas they increased strongly in the cells exposed to UVPAPC, indicative of ongoing lipid peroxidation.

PL hydroxides were elevated immediately after UVA exposure and remained elevated at the 24 h timepoint. UVPAPC treatment resulted in high levels of PAPC-OH (contained in UVPAPC) which were further increased at 24 h. We observed immediate generation of PLPC-OH in response to UVA, less so by UVPAPC, but both treatments readily induced PLPC-OH 24 h after exposure. These data indicate that the reduction of PL-OOH and/or the synthesis of PL-OH induced by UVPAPC exposure were slower than that evoked by UVA exposure (Fig. 2F, J).

Phospholipid oxidation can however yield a broad spectrum of other products that result from cyclization or oxidative fragmentation of the polyunsaturated fatty acid chain [10], giving rise to bioactive isoprostane-, fragmented carbonyl- or di-carboxylic acid containing PLs, as prominent examples. The kinetics of 1-palmitoyl-2-(5-oxovaleroyl)-*sn*-glycero-3-phosphocholine (POVPC, carbonyl) and 1-palmitoyl-2-glutaroyl-*sn*-glycero-3-phosphocholine (PGPC, acid), both products of PAPC (Fig. 2G,H) show that UVA exposure of KC leads to an immediate rise in these species, but that they return to baseline after 24 h. The same was observed for the fragmented products 1-palmitoyl-2-(9-oxo)nonanoyl-*sn*-glycero-3-phosphocholine (PONPC, carbonyl) and 1-palmitoyl-2-azelaoyl-*sn*-glycero-3-phosphocholine (PAzPC, acid) derived from PLPC (Fig. 2K, L). UVPAPC supplementation led, as anticipated to an immediate rise in PAPC derived fragmented species which amplified at 24 h. UVPAPC supplementation did however not lead to an immediate rise in PLPC derived fragmented species, and at 24 h to a moderate elevation of PONPC but not PAzPC. Quantification of the corresponding lipids with stearic instead of palmitic acid in the sn-1 position which yielded comparable results, and quantification of the unoxidized precursors, isoprostanoid- and lysophospholipid species are shown in Supplementary Fig. 2.

Thus, this first analysis of oxPC species in UVA- stressed human keratinocytes demonstrated that fragmented PC oxidation products, among them reactive aldehydophospholipids, are immediately elevated by UVA, but efficiently restored to baseline level within 24 h, even when there is ongoing lipid peroxidation, as shown by still elevated levels of PL-OOH and OH at 24 h.

Further, oxidized lipid stress as elicited by addition of external UVPAPC, does, with delay induce peroxidation of unrelated PL species shown by PLPC-OOH formation kinetics. However, either the formation of fragmented products of unrelated PLs (PAzPC, SONPC, SAzPC) is selective or prevented by induced cellular responses.

### 2.3 A high-resolution MS method identifies uncharted UV-generated phospholipids

**Fig. 3:**
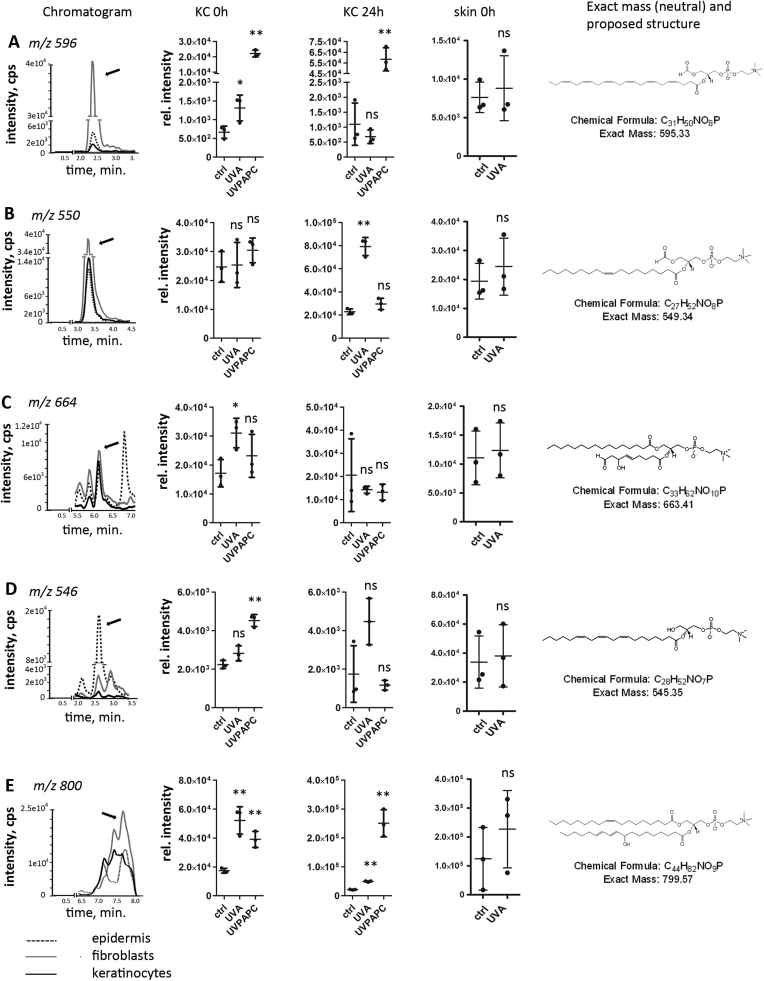
**High resolution MS identifies uncharted UV-generated phospholipids.** Panels **A-E** show first the extracted ion chromatograms (XIC) of the respective multiple reaction monitoring (MRM) transitions in lipid extracts from cultured human keratinocytes (solid black line), human fibroblasts (grey line) and human total epidermal lipid extracts (dotted line). Next, dot blots show abundance of the respective species in cultured KC at 0 h and 24 h post stress and in lipid extracts from unirradiated and *ex-vivo* irradiated human skin samples(n = 3; error bars indicate SD). At the right side of each panel, the chemical formula, the exact mass and a proposed structure as determined by the high-resolution MS/MS method are presented. **A,***m/z* 596 – PC (22:6, C1 carbonyl); **B**, *m/z* 550 – PC (18:1, C1 carbonyl); **C**, *m/z* 664 – PC (16:0, C9 carbonyl, monohydroxy, one double bond); **D**, *m/z* 546 – PC (20:3, lyso); **E**, *m/z* 800 – PC (18:1, 18:2, monohydroxy). Asterisks indicate significant differences (*P < 0.05; ** P < 0.01) determined by Student's *t*-test.

Besides these identified species, the bulk of the UV regulated lipid signals could however not be unambiguously identified by our screening method. Thus, 20 analytes were selected for further analysis based on the criteria that they were highly inducible by UVA at 0 h or 24 h and that they were detectable also in human epidermis or in dermal fibroblasts. We used an independent high-resolution MS/MS approach combining data from positive and negative ionization modes. Precursor ions corresponding to the previously identified SRM transitions and collision-induced dissociation (CID) analysis in positive ion mode confirmed the presence of phosphatidylcholine fragment ion at *m/z* 184.1. Negative ion mode tandem mass spectra allowed identification of fatty acid composition for modified lipids. Using this approach, we propose structures for five UVA regulated oxidized PCs (Fig. 3A-E and Supplementary Fig. 3A-E).

Based on the tandem mass spectra for protonated ions at *m/z* 596.33 (Fig. 3A, Supplementary Fig. 3A), 550.35 (Fig. 3B and Supplementary Fig. 3B), and 664.42 (Fig. 3C and Supplementary Fig. 3C), carbonyl group containing structures were proposed. The signal at *m/z* 596.33 which we propose as PC carrying docosahexaenoic acid and C1 terminal carbonyl was highly inducible by UVPAPC immediately and after 24 h, by UVA only immediately after exposure. The signal at *m/z* 664.42, proposed as PC (16:0_9:1) with C9 terminal aldehyde and hydroxy group within the same fatty acid chain, was increased immediately after UVA exposure but not by UVPAPC stress. The signal at *m/z* 550.35 corresponding to PC with oleic acid and C1 terminal carbonyl exclusively increased 24 h post UVA exposure, thereby having a strikingly different kinetic than all other aldehyde species described here. The lysoPC (20:3) at *m/z* 546.36 (Fig. 3D, Supplementary Fig. 3D) strongly increased immediately after addition of UVPAPC but returned to baseline level after 24 h. Finally, the proposed hydroxy derivative of PC (18:1_18:2) at *m/z* 800.58 (Fig. 3E and Supplementary Fig. 3E) had kinetic similar to the other PL-hydroxides described in Fig. 2. We verified the presence of all five newly identified oxidized lipid species in human skin explant biopsies where no significant changes in the relative abundance were observed immediately after UVA (80 J/cm^2^) exposure (Fig. 3A-E; right data panel).

### 2.4 UVA and UVPAPC effects on the KC transcriptome

To identify candidate genes, pathways or higher order regulators that may be involved in regulating the partial restoration of lipid oxidation homeostasis after redox stress, we performed transcriptomic and proteomic experiments followed by bioinformatic analysis.

**Fig. 4:**
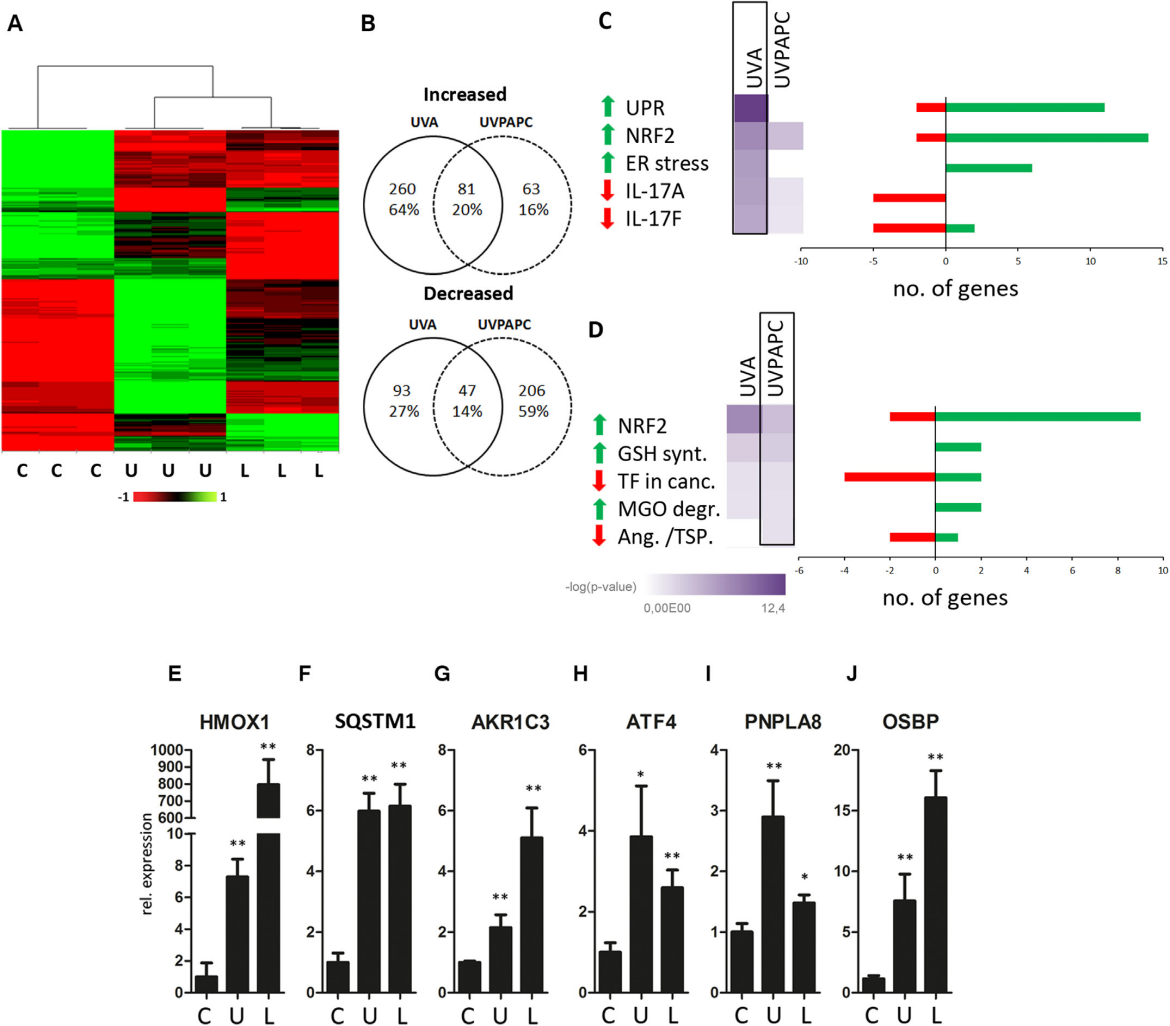
**UVA and UVPAPC effects on the KC transcriptome.** Human primary keratinocytes were treated with UVA (“U”; 40 J/cm^2^), UVPAPC (“L”; 25 µg/ml) or were sham treated (“C”). 7 h after the stress treatment total RNA was isolated from biological triplicates and global gene expression was analyzed with microarray technology. Genes that showed significant regulation (p < 0.05; moderated t-statistics (LIMMA) with Benjamini-Hochberg correction) of > 2fold were used for further analysis. **A** Heat map analysis of gene expression. Columns represent the different treatments; rows show the different Gene IDs of mRNAs (color code: green, up-regulation; red, down-regulation). **B** Venn diagrams showing the number of regulated genes (up- and down-regulated, respectively) that overlapped between the stress treatments. **C,D** Top five pathways of the canonical pathway analysis using IPA software in UVA-(C) or UVPAPC exposed cells (D) are listed, bar graphs indicate number of up- (green) or down- (red) regulated genes within each term. Heatmaps indicate p-values. **E-J** Verification of highly regulated genes within the top canonical pathways Nrf2 (Heme oxygenase 1 (HMOX1),E; Sequestosome 1 (SQSTM1), F; and aldo-keto reductase 1 C3 (AKR1C3), G); the unfolded protein response pathway (UPR) (ATF4, H) and lipid modifying genes (again AKR1C3, G; patatine-like phospholipase A8 (PNPLA8), I; and oxysterol binding protein (OSBP), J), respectively, using qPCR (n = 3; relative quantification, normalized to expression of beta-2-microglobulin; error bars indicate SD). Asterisks indicate significant differences (*P < 0.05; ** P < 0.01) determined by Student's *t*-test.

UVA irradiation significantly increased the expression of 341 genes more than two -fold and led to a down-regulation of 140 genes. Treatment with UVPAPC increased expression of 143 genes and led to a decrease of 253 genes, respectively (Fig. 4A, B and Supplementary Table 1). The Venn diagrams show the number of genes whose regulation overlapped upon both treatments. Of the UVPAPC regulated genes, 81 were co-induced, whereas 47 were co-decreased upon UVA exposure. To visualize the mRNA expression pattern of the stressed cells compared to the controls we performed a principal component analysis (Supplementary Fig. 4A) which together with the heatmap (Fig. 4A) confirmed reproducibility within- and clear separation between the treatment groups.

We used “Ingenuity Pathways Analysis” software (IPA, Qiagen) to investigate whether the regulated gene groups would allow predicting activation or inhibition of canonical signaling pathways. The pathway analysis indicated that both stressors, UVA (Fig. 4C) and UVPAPC (Fig. 4D), significantly induced the oxidative stress response controlled by NRF2 (Gene Heatmaps Supplementary Fig. 4B). Additionally, UVA significantly induced the unfolded protein response (UPR; Supplementary Fig. 4C)- and the endoplasmic reticulum (ER) stress pathway and inhibited interleukin 17 A and – F signaling (Fig. 4C). UVPAPC treatment activated, in addition to NRF2, genes attributed with functions in glutathione biosynthesis or methylglyoxal degradation and reduced the “role of tissue factor in cancer” and the “inhibition of angiogenesis by TSP1” pathways (Fig. 4D).

We verified regulation of NRF2 dependent gene expression by qPCR for HMOX1 (Fig. 4E), the autophagy adaptor sequestosome 1 (SQSTM1, p62; Fig. 4F) and AKR1C3 (Fig. 4G), and for the UPR marker ATF4 (Fig. 4H). As the dynamic changes in the oxidized phospholipidome over time suggest the involvement of inducible lipid metabolizing enzymes, we screened for candidates carrying the string “lipid” in their gene ontology (GO) database entry for biological function (Supplementary Table 2A,B). Exemplary lipid metabolism genes that were induced both by UVA and UVPAPC were aldo-keto reductase 1C family genes (AKR1C3), patatin-like phospholipase domain containing 8 (PNPLA8, calcium-independent phospholipase A2) and the oxysterol binding protein (OSBP) with potential roles in the signaling, detoxification and degradation of reactive oxidatively modified (phospho)lipids (Fig. 4 G, I, J). Volcano plots with highlighted members of the NRF2, UPR and “Lipid” groups are provided in Supplementary Fig. 6 (A-D).

### 2.5 UVA and UVPAPC effect on the KC proteome

Next, we investigated whether the overlapping responses to UVA and UV-oxidized lipids also would be apparent at the proteome level 24 h after treatment.

**Fig. 5:**
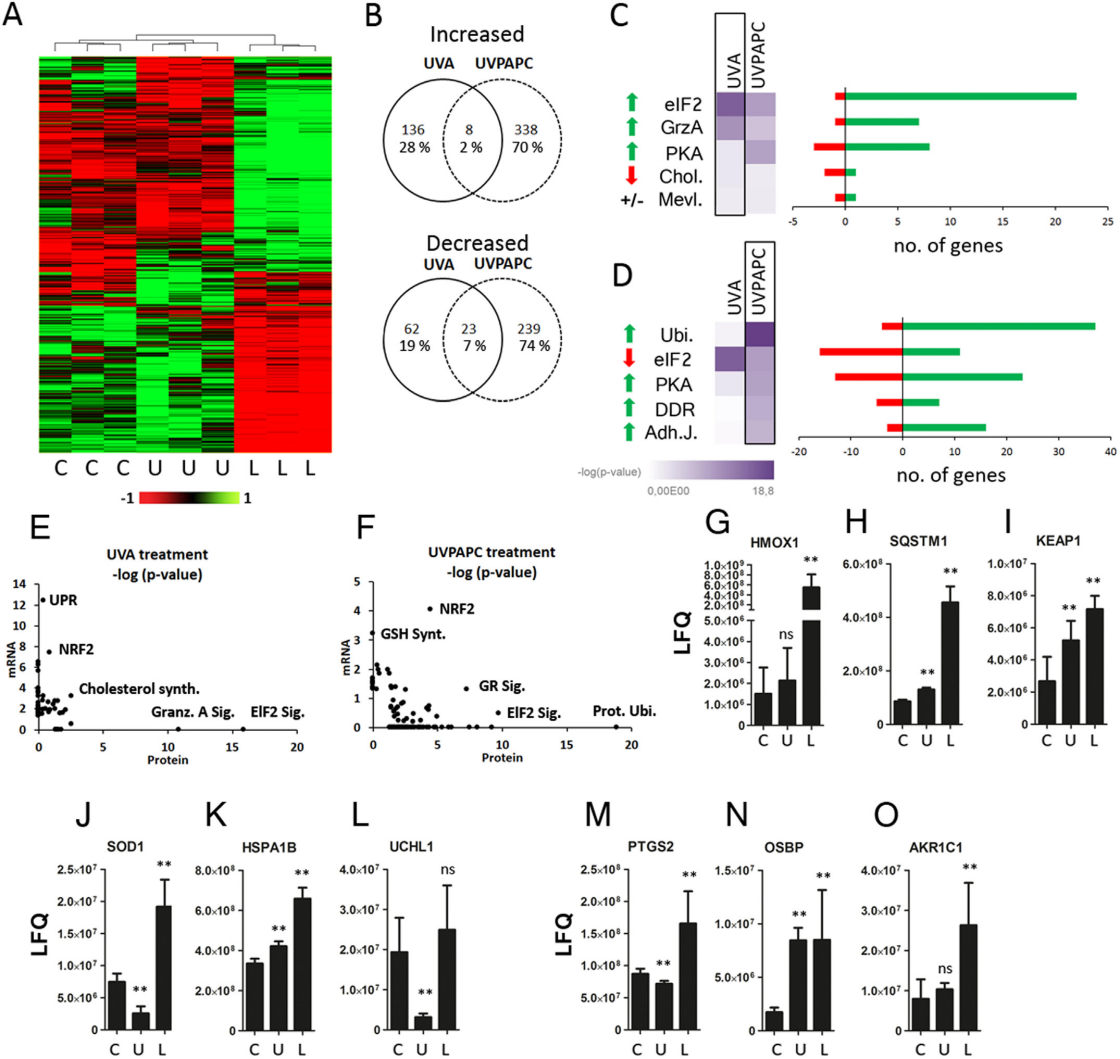
**UVA and UVPAPC effect on the KC proteome.** Purified proteins of human KC 24 h post stress treatment (“U”, UVA-1 40 J/cm^2^ and “L”, UVPAPC 25 µg/ml, respectively) were analyzed by LC-MS/MS based label free proteomics. Experiments were performed in biological triplicates. Proteins that showed significant regulation (p < 0.05; two-sided *t*-test with permutation based- false discovery rate “FDR”) of > 1.5 fold were used for further analysis **A** Heat map analysis. Columns represent the different treatments; rows show the different protein IDs (color code: green, up-regulation; red, down-regulation) **B** Venn diagrams showing the number of regulated proteins that overlapped between the stress treatments. **C,D** Top five hits of the canonical pathway analysis in UVA-(C) or UVPAPC exposed cells (D) are listed. Next to the heatmaps bar graphs indicate the number of up- (red) or down- (green) regulated genes assigned to the respective pathways. **G-O** Bar graphs depict the regulated proteins 24 h after stress treatment label free quantification (LFQ) values. Asterisks indicate significant differences (*p < 0.05; ** p < 0.01) determined by Student's *t*-test.

UVA significantly increased the abundance of 144 proteins more than 1.5 -fold whereas 85 proteins were downregulated after 24 h. After the UVPAPC treatment 346 proteins were found significantly increased and 262 proteins were decreased (Fig. 5A, B; Supplementary Table 3; PCA analysis in Supplementary Fig. 5A). Venn diagrams (Fig. 5B) show the co-regulation of thirty one proteins (8 up-, 23 down-regulated, respectively) by the two treatments after 24 h, which is less (2% and 7%, for increased and decreased proteins, respectively) than on mRNA level. We investigated activation or inhibition of canonical signaling pathways with IPA software, and found proteins associated with eIF2 stress signaling and protein kinase A (PKA) pathway activated, additionally a protein ubiquitination and DNA damage response and epithelial adherens junction signaling was regulated upon UVPAPC (Fig. 5C, D).

Comparing the changes on mRNA level at 7 h post treatment to the effects on the proteome at 24 h post stress, we observed an overlap of only 12 co-regulated mRNAs and proteins for UVA, and 18 for UVPAPC (listed in Supplementary Table 4). Discrepancies between mRNA and protein changes upon a comparable type of stress have been reported recently [16], so we asked whether there would be functional overlaps in the responses beyond individual mRNAs or proteins. We thus plotted the IPA protein pathways ranked by significance (-log of the p-values) to pathways regulated on mRNA level. Here, it became apparent that the NRF2 system was significantly co-regulated on the pathway level upon UVA or OxPL exposure (Fig. 5E, F and Supplementary Fig. 5B). Importantly, the low overlap in individual genes co-regulated on mRNA and protein level was contrasted by a large functional overlap, as the UPR signature on mRNA level was matched by a downstream eIF2 signature on protein level in the UVA treated cells (Fig. 5E and Supplementary Fig. 5C). A lipid metabolic pathway (cholesterol synthesis) was significantly enriched in both proteome and transcriptome after UVA stress treatment (Fig. 5C, Supplementary Table 5). Volcano plots for proteins in the groups NRF2, UPA and “lipid” are presented in Supplementary Fig. 6 (E-H).

In Fig. 5 G-O examples of regulated proteins are presented, among them markers for NRF2 activation and lipid metabolization. Besides HMOX1 also SQSTM1 which is both a NRF2 target and a central regulator of autophagy [39] and KEAP1, the cytoplasmic binding partner for NRF2, were found increased in the stressed KC (Fig. 5G-I). Superoxide dismutase 1 (SOD1), was UVA depleted but induced by UVPAPC at 24 h post stress, whereas the heat shock protein 70 (HSP70) component HSPA1B was moderately but significantly increased by both treatments (Fig. 5J, K). UCHL1, an enzyme regulating the availability of mono-ubiquitin, was strongly decreased by UVA, and UVPAPC led to moderate induction of cyclooxygenase 2 (PTGS2) protein (Fig. 5L, M). In line with the mRNA results, OSBP, a protein regulating intracellular transport of oxysterols [49] was induced by both treatments. AKR1C1, a NRF2 dependent enzyme that reduces carbonyl groups on lipids [12] was effectively induced by UVPAPC exposure (Fig. 5N, O).

### 2.6 Identification of NUPR1 as transcriptional upstream regulator

**Fig. 6:**
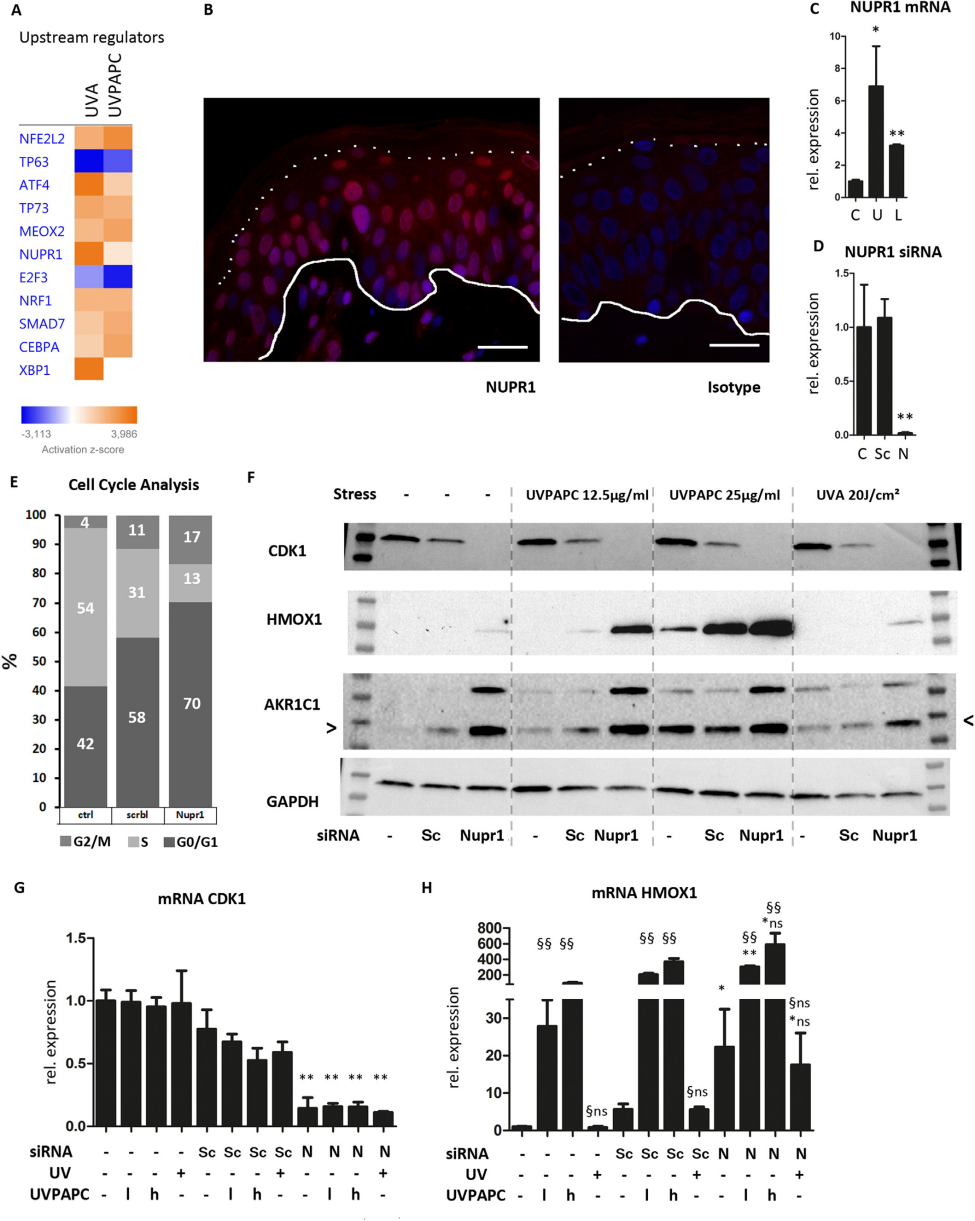
**Identification of NUPR1 as transcriptional upstream regulator. A** Heatmap of the activation *z*-score for the respective proposed upstream regulators after UVA (“U”) -or UVPAPC (“L”) treatment. **B** NUPR1 protein expression in human skin sections (immunofluorescence microscopy, NUPR1: red, Nuclear stain: blue; representative image, scale bar = 20 µm). **C/D** Relative mRNA expression of NUPR1 in cultured primary epidermal KC was quantified by qPCR and normalized to expression of beta-2-microglobulin (n = 3; error bars indicate SD, Asterisks indicate significance treatment vs. ctrl in C and knockdown vs. scramble in D determined by Student's *t*-test; *p < 0.05; ** p < 0.01); “C”, Control; “U”, UVA-1 40 J/cm^2^, “L”, UVPAPC 25 µg/ml). **E** Cell cycle analysis using FITC/ BrdU staining and flow cytometry. Transiently transfected keratinocytes using a scrbl siRNA and siRNAs targeting NUPR1 were analyzed to determine the percentage of cells in the S, G0/G1 or G2/M phases 48 h post transfection. **F,G,H** Keratinocytes that were either non-transfected, scramble siRNA transfected (Sc) or Nupr1 siRNA transfeced (Nupr1 or N) were treated with UVA-1 (40 J/cm^2^) or UVPAPC (12,5 µg/ml “l”, and 25 µg/ml, “h”). 24 h post stress treatment protein levels (F) of CDK1, HMOX1, AKR1C1 (arrows) and GAPDH were assayed by immunoblotting. 7 h after the stress treatment relative mRNA expression of CDK1 (G) and HMOX1 (H) was quantified by qPCR (n = 3; relative quantification, normalized to expression of beta-2-microglobulin; error bars indicate SD). “§” indicate significant differences between treatment and control and “*” between Nupr1 knockdown and equally treated scramble siRNA transfected cells (*,§ p < 0.05 and **,§§ p < 0.01) as determined by Student's *t*-test.

While the NRF2 pathway was thereby confirmed as shared regulator of UV- and lipid responses, we used the “upstream regulators” feature of the IPA software to predict further regulators potentially implicated in KC redox stress responses (Fig. 6A). The algorithm indeed predicted NRF2 as an activated upstream regulator, also ATF4, which regulates UPR-dependent transcription was predicted activated, especially in the UVA treated cells, in line with the previous findings. Another upstream factor predicted to be strongly activated was nuclear protein 1 (NUPR1/p8/COM1), a stress inducible transcriptional regulator [13], [2]. NUPR1 is overexpressed in various malignancies, induced by cellular stress and is an established regulator of autophagy [44]. Expression of NUPR1 has not been previously described in the skin, and we could detect it in the majority of the nuclei of keratinocytes within the living layers of the epidermis (Fig. 6B). The nuclear staining of NUPR1 in the positive cells appeared more intensive in the spinous layer as compared to the positive cells in the basal layer. We found that NUPR1 mRNA itself was significantly induced by UVA and to a lesser extent also by UVPAPC in cultured primary epidermal KC (Fig. 6C). NUPR1 had been associated with functions in autophagy and MAPK signaling [29] which are both activated by OxPL. We suppressed NUPR1 in KC with stealth siRNA transfection (Fig. 6D) with no obvious effect on cell morphology and viability (Supplementary Fig. 7). Cell cycle analysis revealed an increased percentage of cells in G0/G1 and G2/M phase upon NUPR1 knockdown (G0/G1: 70% vs. 58%; G2/M: 17% vs. 11%) and a lower percentage of cells in the S phase (13% vs. 31%). This stop in cell cycle progression was in line with a decrease of cyclin dependent kinase 1 (CDK1) in NUPR1 knockdown cells on mRNA and protein level, respectively (Fig. 6F, G). We next investigated how the knockdown would affect target genes and proteins which are stress regulated and have been associated with NUPR1 in other cell types, most prominently HMOX1 [14], [78]. Indeed, we found on protein and mRNA level (Fig. 6F, H) that HMOX1 was up-regulated in NUPR1 knockout cells compared to scramble transfected cells. On protein level we observed highly elevated expression of HMOX1 in NUPR1 knockout cells after treatment with UVPAPC. The knockdown of NUPR1 led to a strongly increased basal AKR1C1 protein expression (Fig. 6F), as one example of a lipid detoxification gene. As the several of the genes affected by NUPR1 knockdown are known Nrf2 targets, and as NUPR1 is stress regulated, we investigated we investigated their interdependence. Knockdown of NUPR1 did not significantly alter expression or nuclear translocation of NRF2 (Supplementary Fig. 8). NRF2 knockdown did not affect NUPR1 baseline or UVPAPC induced expression, but blunted stress induced expression of HMOX1 and AKR1C3, compatible with a model in which NUPR1 requires functional NRF2 for target gene induction (Supplementary Fig. 9). The interdependence of the two transcriptional regulators requires however further investigation in gene deficient systems that omit lipofection, as the lipofection process possibly causes stress that affects these pathways. NUPR1 contains amino acids that potentially allow modification by electrophilic reactive compounds, thus we investigated whether NUPR1 protein would be modified in vitro by oxidized lipids. We incubated recombinant, purified GST tagged NUPR1 protein (NUPR1-GST) with increasing doses of oxidized PAPC or the non-oxidized saturated 1,2-dipalmitoyl-*sn*-glycero-3-phosphocoline (DPPC) as a control. We also added the singlet oxygen quencher sodium azide NaN3 or the antioxidant butylated hydroxytoluene (BHT) to the reaction mixture. We then separated the incubation mixtures on an acrylamide gel and performed Western blot. Using an anti-NUPR1 antibody we observed that high molecular weight (HMW) aggregates were formed with increasing doses of UVPAPC (Supplementary Fig. 10, checkered arrows). Incubation with non-oxidized DPPC had no major effects on NUPR1 electromobility. While incubation in presence of 1 mM NaN3 reduced HMW aggregates, we observed an additional band at 150 kDa (black arrow). Incubation in the presence of 0001% w/v BHT failed to inhibit the formation of oxidized PAPC - HMW aggregates. While we cannot rule out that the GST tag contributed to the observed effects, this is to our knowledge the first time that an interaction of oxidized PAPC with a specific protein is shown to stably modify or crosslink it. In addition, the high molecular weight products can be partially inhibited by sodium azide, but not by the antioxidant BHT.

## 3 Discussion

In this study we determined the contribution of bioactive lipids to the responses of epidermal keratinocytes to UV irradiation, the most relevant extrinsic stressor of the skin. We focused on oxidized phosphatidylcholines, which are increasingly recognized as signaling molecules [8]. The semi-targeted HPLC MS/MS screening approach [31] revealed regulation patterns for known and unidentified lipid species. We investigated the latter with a high precision MS method to identify the exact mass and propose potential structures. Using transcriptomic and proteomic profiling, we identified candidate genes and proteins potentially involved in signaling, metabolization, detoxification and *de novo* synthesis of selected lipid mediators. We identified NUPR1 as so far unknown upstream regulatory factor in the shared response to UV and oxidized lipids of the skin. The knockdown of NUPR1 indeed affected critical UV responsive genes governing the antioxidant response, lipid detoxification, autophagy and cell cycle and thus suggests NUPR1 to be a central, lipid regulated orchestrator of cutaneous stress responses.

Among the lipid species we found significantly regulated were several species previously identified in autoxidized preparations of their unoxidized precursors [31], [58], [72], but also previously undescribed species. We will now discuss the regulated lipids or lipid classes, their potential biological relevance and how the regulated genes and proteins may act as effectors or degraders of UV generated lipids.

All four quantified phospholipid hydroperoxides were, immediately elevated in UVA- exposed and also in UVPAPC- treated keratinocytes. Typically, the PL-OOH are reduced to PL-OH, and we found immediate rise in PC-OH after UVA exposure, while the PLPC-OH peaked 24 h after the stress elicited with externally added UVPAPC. A similar kinetic was observed for the newly discovered UVA- and UVPAPC regulated lipid species at *m/z* 800 (proposed structure 1-oleoyl-2-linolenoy-PC hydroxide). This indicates that under UVPAPC-induced stress additional, probably enzymatic, generation of PL-OH was favored. Indeed, with PRDX6 and glutathione peroxidases, we found enzymes induced that catalyze the reduction of PLPC-OOH to –OH. While low levels of PL-OOH are permanently formed in metabolism, increasing concentrations initiate apoptotic signaling and very high levels cause structural membrane damage and necrotic cell death [23], [28]. We could recently associate PRDX6 expression with epidermal PL hydroperoxide levels in the mouse [60], and our new findings strongly support that this is also the case in humans. Lipid species previously assigned as isoPGF2a modifications of PAPC and SAPC were induced weakly by UVA, strongly by UVPAPC, and increased further at 24 h. Isoprostanoid modifications of PAPC were described as most efficient inducers of UPR genes [27] via activation of the transcription factor ATF4 [55]. Individuals with increased skin photoaging have reportedly higher levels of plasma isoprostanes [15] and as a result of UVB exposure [66].

Moving from chain-intact oxygenated species to oxidatively fragmented species, we found carbonyl- and dicarboxylic acid containing species regulated, and identified three previously undescribed species with a carbonyl modification. POVPC and PONPC, aldehydo-PL oxidation products of PAPC and PLPC, respectively [8], were augmented immediately upon UVA exposure but declined to baseline levels after 24 h, as did the respective di-carboxylic acid PC species derived from the same precursors. Accordingly, UVPAPC-induced di-carboxylic acid PC species of PLPC (PAzPC), SLPC (SAzPC) and SAPC (SGPC) returned to baseline after 24 h (while PAPC -derived PGPC remained strongly elevated). With *m/z* 596 we propose a 22:6, C1 carbonyl -PC that is inducible by both stimuli, returns to baseline 24 h post UVA, and with *m/z* 664 we found one UVA inducible species with structural similarity to 4-HNE that also returns to baseline after 24 h. The fragmented carbonylic species, the best studied being POVPC exert, apart from their potentially detrimental chemical reactivity, potent signaling functions that require tight control of their bio-availability. POVPC disrupts the endothelial barrier in lung vessels [7], activates the NLRP3 inflammasome [83], has PAF-agonistic activity, and thus together with structurally similar alkyl phospholipids are critical regulators of UV induced immunomodulation and photosensitivity [82]. Together, the data show that KC can restore basal levels of UVA-induced fragmented lipid species within 24 h, with the exception of a newly discovered at *m/z* 550, proposed to be 18:1; C1 carbonyl PC that warrants further biological investigation. Finally, we discovered a regulated lysophospholipd species, 20:3 lysoPC (*m/z* 546), which was elevated by UVPAPC immediately after treatment but disappeared at 24 h. This lysophospholipid had been found previously in human plasma [19], and was released by oncogene induced senescent cells into extracellular vesicles [11], which may also present a way to modulate the cellular levels of specific lipid species and could also provide novel lipid members of the senescence associated secretory phenotype (SASP) in addition to those we recently found in melanocytes [53].

In line with our previous findings in fibroblasts, the transcriptomes of UVA- and UVPAPC stressed cells revealed a substantial overlap that mainly reflected induction of NRF2 dependent genes and genes involved in lipid metabolism. The major divergence in the transcriptional patterns was the induction of UPR / ER-stress genes 7 h after UVA exposure. The proteomic study we performed, in our knowledge the first conducted on UV exposed primary keratinocytes, also identified NRF2 targets upregulated, and UPR induction (pathways UPR, protein ubiquitination and EIF2). Of note, the overlaps between UVA and UVPAPC regulation, but also between mRNA and protein regulation were mostly observed on the pathway rather than on the individual gene level, as found by others in comparable settings [16].

The investigation of upstream regulatory factors confirmed activation of the NRF2 and ATF4 pathways and additionally uncovered NUPR1 as central factor affecting antioxidant response, lipid detoxification, cell-cycle- and autophagy genes. NUPR1 is regulated by pattern recognition receptors (PRR) activation [40], and OxPL are known ligands for TLR2 [42] but also antagonists of TLR4 [9]. NUPR1 induction was observed upon ER stress [56] and the ER stress sensitive transcription factor ATF4 induces NUPR1 in starvation [3] and toxic stress [25]. ATF4 is activated by oxidized PAPC [55] downstream of NRF2 [1], and is also induced transcriptionally by UVA and UVPAPC in our keratinocyte system. HSPA5/GRP78, a receptor for OxPAPC, is both a target gene of ATF4 and its transcriptional activator [6]. ATF 4 activation via NRF2 and/or GRP78 is thus the most likely mechanism for lipid- and redox stress mediated NUPR1 regulation in epidermal cells. The reported downstream effects of NUPR1 suggest cell- and tissue dependence of its function in stress mediated proliferation and metabolism control. NUPR1 knockdown in glioblastoma cells suppressed cell growth [48], by repressing ERK1/2 and p38 MAPK phosphorylation, two signaling pathways inducible by OxPAPC [5]. A further possible route by which NUPR1 attenuated cell cycle progression was via p53/p21 regulation [17]. NUPR1 was downregulated in sebocyte tumors which derive from epidermal appendages, and was identified as a target of Rac1 GTPase [24]. Regarding stress mediated changes in metabolism, inactivation of NUPR1 increased autophagy in cardiac myocytes [44] and neuronal cells experiencing ER stress [81]. As we have recently shown that autophagy is induced by UVA/UVPAPC in keratinocytes [85], and that genetic deletion of autophagy results in accumulation or secretion of selected oxidized lipid mediators in cutaneous cells [53], [84], further studies will address the contribution of NUPR1 to epidermal autophagy. We for the first time demonstrate that exposure to oxidized PL can modify and most likely crosslink a recombinant, tagged form of NUPR1. As OxPL treatment and NUPR1 knockdown both result in induction of HO-1 and AKR1C1, we propose that UV/OxPL induced modification could make NUPR1 unavailable to exert repression of these genes. It will be of interest to further follow the hypothesis that NUPR1, possibly in coordination with NRF2, maintains defense mechanisms of the epidermis an autoregulatory fashion through reactive lipids generated by stress or in differentiation, and how such a system would adapt during the chronological- and photo aging process, where an accumulation of the reactive lipid in the tissue would be expeced as a result of decreased redox surveillance of the cells.

Taken together, our data add novel aspects to the knowledge on the cutaneous responses to lipid oxidizing stress. The lipid mediators induced by long wave UV light have both signaling function and chemical reactivity towards proteins. We detected redox stress responses (NRF2, ATF4/UPR, lipid metabolizing enzymes), which are likely to contribute to the restoration of lipid homeostasis. NRF2 directed glutathione de-novo synthesis and recycling is essential for PL-OOH reduction, and the identified aldo-keto reductases can reduce lipid aldehydes. Further, phospholipases that cleave arachidonic acid from the PC were induced by both treatments (PNPLA8) and PAFAH1B3 which specifically cleaves fragmented OxPL [36] and PAFAH prevent lipotoxicity in response to UV stress [50]. Of note, peroxiredoxin 6 (PRDX6) which has lipid hydroperoxide reductase- lysophosphatidylcholine acyl transferase and PLA2 function [21] was induced by UVPAPC, adding to our previous findings on its role in epidermal lipid homeostasis [60]. With the bioinformatic identification and functional verification of NUPR1 we put forward a novel factor controlling epidermal cell growth and redox defenses relevant in homeostasis, aging and disease.

## 4 Materials & methods

### 4.1 Primary keratinocyte culture and skin biopsies

Human neonatal primary keratinocytes were received from Cell Systems (Troisdorf, Germany) or keratinocytes were prepared from abdominal adult skin from obtained from plastic surgery as described previously [51]. Cells were cultured in serum-free keratinocyte growth medium (KGM-2, Lonza, Basel, Switzerland) at 37 °C and 5% CO_2_ for further treatments. The collection of the biopsies used for immunohistological analysis used in this study was approved by the Ethic Committee of the Medical University of Vienna (1149/2011) and written informed consent was obtained from all subjects.

### 4.2 Stress treatment

Cultured KC were irradiated with UVA-1 (340–400 nm) emitted from a Sellamed 3000 (Sellas, Ennepetal, Germany) device at a distance of 20 cm to achieve a total fluence of 20 J/cm^2^ or 40 J/cm^2^ as measured with a Waldmann UV-meter (Waldmann, Germany), respectively [31]. During the irradiation cells were kept in phosphate-buffered saline (PBS) on a temperature controlled plate at 25 °C. UVPAPC was generated by exposing dry PAPC (Avanti Lipids, Alabaster, Alabama) to UVA-1 with a fluence of 80 J/cm^2^ or sham irradiated as described in [35] and cells were treated with 25 µg/ml of UVPAPC. Skin explants – adult skin obtained from plastic surgery was cut into 3 cm^2^ pieces, floated in PBS and irradiated with 80 J/cm^2^ of UVA-1, or were sham irradiated to serve as control.

### 4.3 siRNA transfection

Three Stealth siRNAs specific for Nupr1, two stealth siRNAs specific for Nrf2 and a scrambled control were obtained from Invitrogen (Carlsbad, CA). RNA duplex sense sequences used for Nupr1 were: 5′- CCUCUAAGCCUGGCCCAUUCCUAC -3′; 5′- CCGGAAAGGUCGCACCAAGAGAGAA -3′; 5′- GGCACGAGAGGAAACUGGUGACCAA -3′; for Nrf2; 5′- UAUUUGACUUCAGUCAGCGACGGAA -3′; 5′- GAGCAAGUUUGGGAGGAGCUAUUAU -3′; and the Medium GC content negative control siRNA: 5′ – GAGUGGGUCUGGGUCUUCCCGUAGA -3′. At 50–60% confluence keratinocytes were transfected using Lipofectamine 2000 (Invitrogen). 5 ml OPTI-MEM medium (Thermo Fisher Scientific; Waltham, MA) was mixed with 50 μL Lipofectamine 2000 and 60 μL of a 20 μM siRNA solution (20 μL per Nupr1 siRNA; 30 μL per Nrf2 siRNA) or the scrambled control RNA solution. The solution was incubated at room temperature for 30 min and then added to 20 ml KGM-2 and transferred to the KCs. 24 h after incubation cells received new KGM-2 for another 24 h before stress treatment.

### 4.4 Cell viability assay

After a recovery time of 48 h upon transfection cells were trypsin digested and prepared as single-cell suspension in a PBS solution. Cell viability was determined by cell counting in the LunaFL cytometer (Logos Biosystems, Annadale, VA) using an Acridin Orange (AO)/ Propidium Iodide (PI) staining system (AO/PI cell viability kit, Biozym) according to the manufacturer's protocol. Cells that are positive for the cell permeable nucleic acid dye AO but negative for the late apoptotic and necrotic cell marker dye PI, were counted as viable.

### 4.5 Immunofluorescence analysis

Human skin obtained from plastic surgery was fixed with 10% formalin, paraffin embedded and microtome sections (4 µm) were immuno-stained. Primary keratinocytes were fixed with 4% paraformaldehyde (10 min rt) and the permeabilized with PBS containing 0,1% Trition X-100 (Sigma-Aldrich; MO, USA). Sections were incubated overnight at 4 °C in phosphate-buffered saline (pH 7.2, 2% BSA) with the primary antibody Nupr1 (bs-7106R Bioss; 1:500; rb) or Nrf2 (ab62352 Abcam; 1:200; rb). As secondary antibody goat anti-rabbit IgG (H+L), conjugated with Alexa Fluor dyes (Molecular Probes Eugene, OR, USA) were used at a dilution of 1:500. For imaging, an Olympus (Tokyo, Japan) AX 70 was used. All image analyses were performed under the same parameter settings. Nuclear intensity of NRF2 was quantified using ImageJ software.

### 4.6 Cell cycle analysis

Cell cycle analysis was performed using the BrdU cell-cycle kit (containing the reagents below; BD Biosciences, Franklin Lakes, NJ) according to the manufacturer's instructions. 48 h post transfection, cells were incubated with BrdU (10 µM) for 4 h. After fixation with 100 μL of BD Cytofix Cytoperm Buffer for 15 min at 4 °C cells were stained with a fluorescein isothiocyanate (FITC) conjugated anti-BrdU antibody for 30 min and stained with 7-AAD and immediately analyzed on a FACS-Calibur (BD Biosciences). Gates were set according to the manufacturer's instructions and data were evaluated using FlowJo software (Tree Star, Ashland, OR).

### 4.7 Lipid isolation and analysis

Cell culture- Immediately after stress treatment (timepoint 0 h) or after a recovery time of 24 h keratinocytes were washed with PBS containing DTPA (0.5 mM). Keratinocytes from two wells of a 6 well culture dish (together 18 cm^2^, 1,8 × 10^6^ cells) were scraped on ice in 1 ml of methanol/acetic acid (3%)/BHT (0.01%) to obtain material for lipid extraction.

Skin explants- 3 cm^2^ pieces of skin (UVA or sham treated, respectively) were cut into small pieces and incubated for 1 h at 37 °C in dispase II to separate epidermis from dermis. To dissolve the epidermis it was transferred to Precellys tubes with 2 ml of ice cold methanol/acetic acid (3%)/BHT (0.01%) and was shaken 2 times with 5500 rpm for 30 s, centrifuged for 10 min (3000 rpm) and the supernatant was transferred into a glass tube.

Phospholipid isolation – Isolation of lipids from cell culture or skin explants was performed using liquid–liquid extraction procedure, as recently described in Gruber et al. (2007). In brief, the experiment was performed on biological triplicate samples and each step was performed on ice. 10 ng of internal standard (1,2-dinnanoyl-*sn*-glycero-3-phosphocholine [DNPC] (Avanti Lipids) was added into each sample. After washing the samples 3 times with 4 ml hexan/BHT (0.01%), 4 ml chloroform/BHT (0.01%) and 1.5 ml formic acid (0.7 M) were added to the methanol phase and after vortexing the lower organic phase was transferred into a new glass vial, dried under argon and stored at −20 °C until mass spectrometry analysis.

### 4.8 Phospholipid HPLC MS/MS

Analysis of purified phospholipids was performed at FTC-Forensic Toxicological Laboratory, Vienna as recently described by us [31]. In brief, purified samples were reconstituted in 85% aqueous methanol containing 5 mM ammonium formate and 0,1% formic acid. Aliquots (10 μL) were injected onto a core–shell type C18 column (Kinetex 2.6 µm, 50 mm 3.0 mm ID; Phenomenex, Torrance,CA) kept at 20 °C and using a 1200 series HPLC system from Agilent Technologies (Waldbronn, Germany), which was coupled to a 4000 QTrap triple quadrupole linear ion trap hybrid mass spectrometer system equipped with a Turbo V electrospray ion source (Applied Biosystems, Foster City, CA). Detection was carried out in positive ion mode by selected reaction monitoring (SRM) of 99 MS/MS transitions using a PC-specific product ion (*m/z* 184), which corresponds to the cleaved phosphocholine residue. Data acquisition and instrument control were performed with Analyst software, version 1.6 (Applied Biosystems). Individual values were normalized to the intrinsic DPPC.

### 4.9 High-resolution MS

#### 4.9.1 RPLC-MS

Acquity UPLC M-class (Waters GmbH, Eschborn, Germany) was coupled online to a Synapt G2-Si mass spectrometer equipped with an ESI source (Waters GmbH, Eschborn, Germany) operating in negative ion mode. Eluent A was a mixture of water and acetonitrile (90:10, v/v) containing formic acid (0.1%, v/v), and eluent B was a mixture of isopropanol, acetonitrile, and methanol (60:20:20, v/v/v) containing formic acid (0.1%, v/v). Lipids (1 μL in 50% B; each sample in triplicate) were loaded onto a C_18_-column Acquity UPLC® CSH™ C18, (internal diameter 1.0 mm, length 100 mm, particle diameter 1.7 µm) and eluted with linear gradients from 50% to 90% eluent B (30 min) and to 99% B (1 min, and held for 10 min). Column temperature was set to 50 °C and the flow rate to 60 μL/min.

Sampling cone voltage was set to 40 V, source offset to 60 V, source temperature to 120 °C, cone gas flow to 30 L/h, desolvation gas flow to 650 L/h, desolvation temperature to 250 °C, nebuliser gas pressure of 6 bar, and an ion spray voltage of −2.0 kV. Data were acquired in negative and positive ion data-dependent (DDA) resolution modes. Precursor ion survey scans (scan time 0.5 s) were acquired for *m/z* 200–1200. Tandem mass spectra (ramp collision energy: LM CE start/end 10–40 and HM CE start/end 20–60) were recorded (scan time 0.25 s) for the 12 most intense signals in each survey scan using a dynamic exclusion for 30 s. The signal of Leu-encephalin (554.26151^-^) was acquired as lock mass [54]. Tandem mass spectra were manually analyzed.

### 4.10 RNA Isolation

#### 4.10.1 Gene array

Total RNA was extracted from human neonatal keratinocytes grown in 12-well culture plates 7 h after stress treatment. Cells were lysed with TriFast Reagent (VWR Peqlab, Radnor, Pennsylvania) according to the manufacturer's instructions. *qPCR*- 7 h after stress treatment total RNA from adult KC were isolated using RNasy 96 system (Qiagen, Hilden, Germany) according to the manufacturer's protocol. RNA quality was assessed with Agilent 2100 Bioanalyzer (Agilent Technologies, Santa Clara, CA) and RNA integrity numbers (RIN) were determined. Samples with a RIN number above 9.0 were used for transcriptomic analysis.

### 4.11 Microarray

Total RNA cleanup and concentration was performed using the RNeasy MinElute Cleanup Kit (Qiagen) according to the manufacturer's recommendations. 200 ng of each sample were used for gene expression analysis with Affymetrix (Sta. Clara, CA) human PrimeView 3`IVT. Hybridization and scanning were performed according to manufacturer's protocol (http://www.affymetrix.com). The experiment was performed on biological triplicate samples. The full microarray data was uploaded to the Gene Expression Omnibus (GEO) with the identifier GSE104870.

### 4.12 Quantitative PCR

400 ng of isolated RNA was reverse transcribed using iScript cDNA Synthesis Kit (Bio-Rad, Hercules, CA) and was diluted 1:5 for further quantitative PCR (qPCR). LightCycler 480 and the LightCycler 480 SYBR Green I Master (Roche, Basel, Switzerland) was used with a standard protocol described before [32] for qPCR. All primer sequences are shown in Supplementary Table 6. Relative quantification of target genes was performed using beta-2 microglobulin as a reference gene.

### 4.13 Protein isolation and analysis

**Western blot** 24 h after stress treatment human KCs were washed twice with PBS and then harvested with lysis buffer (70 mM Tris-HCl, pH6.8, 1,1%SDS, 11,1% (v/v) glycerol, 0005% bromophenol blue (BioRad)) containing protease inhibitor cocktail (Abcam, Cambrige, UK) and Pierce TM Phophatase Inhibitor Mini Tablets (Thermo Fisher Scientific, Waltham, MA) on ice and immediately sonicated. Immunoblotting using antibodies for CDK1 (1:1000; ab32384, Abcam), AKR1C1 (1:1000; ab192785, Abcam), HMOX1 (1:1000, ADI-SPA-896, Enzo), and GAPDH (1:2000; clone 5G4; HyTest Ltd., Turku, Finland), was performed as previously described [34]. As secondary antibody, goat anti-rabbit IgG-HRP (Biorad 170–6515) or sheep anti-mouse IgG-HRP (NA-931-V, GE Healthcare, Little Chalfont, UK) were used and subsequent chemiluminescent quantification on ChemiDoc imager (Bio-Rad) was performed. The signal was measured with Image Lab 4.1 analysis software (Bio-Rad) and target bands were normalized to GAPDH.

### 4.14 Nupr1 immunoblot

150 ng of the recombinant protein Nupr1 (Novusbio H00026471-P01) were incubated with oxPAPC, with or without pretreatment with either 0.001% BHT or 1 mM NaN3 in K2HPO4 in a total volume of 22 ml or were sham treated. 30 min after incubation at 37 °C lysis buffer (70 mM Tris-HCl, pH6.8, 1,1%SDS, 11,1% (v/v) glycerol, 0.005% bromophenol blue (BioRad)) with 5% of Mercaptoethanol was added. Immunoblotting using antibody for NUPR1 (Sigma-Aldrich, 1:1000, rb) was performed as previously described [34] and as detailed above.

### 4.15 Quantitative LC-MS-based proteomics

Cells were washed two times with PBS and scraped in cold PBS 24 h after the stress treatment. The cells were washed again two times with PBS and stored at −80 °C until further analysis. For proteolytic digestion samples were prepared as previously [69]. Briefly, cell pellets were solubilized with urea buffer (7 M urea, 2 M thiourea, 4% CHAPS, 100 mM DTT, 50 mM TEAB and supplemented with protease inhibitors) and sonicated. Protein amounts were estimated with Pierce 660 protein assay. Fifty micrograms of samples were digested with trypsin (1:100 w/w) using the filter-aided sample preparation (FASP) as previously described with minor modifications [80]. Tryptic peptides were recovered, and peptides of protein digests were normalized for tryptophan fluorescence. The peptides were desalted and concentrated with reversed-phase C18 resin. Lyophilized peptides were reconstituted in 5% formic acid and 1 µg of peptides were analyzed by LCMS.

### 4.16 Protein liquid chromatography tandem mass spectrometry

Samples were injected onto a Dionex Ultimate 3000 system (Thermo Fisher) coupled to a Q-Exactive Plus mass spectrometer (Thermo Fisher). Software versions used for the data acquisition and operation of the Q-Exactive were Tune 2.8.1.2806 and Xcalibur 4. HPLC solvents were as follows: solvent A consisted of 0.1% formic acid in water and solvent B consisted of 0.1% formic acid in 80% acetonitrile. From a thermostated autosampler, 10 μL that correspond to 1 µg of the peptide mixture was automatically loaded onto a trap column (PM100-C18 3 µm, 75 µm × 20 mm, ThermoFisher) with a binary pump at a flow rate of 5 μL/min using 2% acetonitrile in 0.1% TFA for loading and washing the pre-column. After washing, the peptides were eluted by forward-flushing onto a 50 cm analytical column with an inner diameter of 75 µm packed with 2 µm-C18 reversed phase material (PepMap-C18 2 µm, 75 µm × 500 mm, ThermoFisher). Peptides were eluted from the analytical column with a 120 min solvent gradient (A: 0.1% FA and B: 80% ACN, 0.1% FA) ranging from 5% to 40% solvent B, followed by a 10 min gradient from 40% to 90% solvent B and finally, to 90% solvent B for 5 min before re-equilibration to 5% solvent B at a constant flow rate of 300 nL/min. The LTQ Velos ESI positive ion calibration solution (Pierce, IL, USA) was used to externally calibrate the instrument prior to sample analysis and an internal calibration was performed on the polysiloxane ion signal at *m/z* 445.120024 from ambient air. MS scans were performed from *m/z* 380–1800 at a resolution of 70,000. Using a data-dependent acquisition mode, the 20 most intense precursor ions (+2 to +6 charge) were isolated (1.6 *m/z* window) and fragmented to obtain the corresponding MSMS spectra. The fragment ions were generated in a higher-energy collisional dissociation (HCD) cell (NCE of 27%) with first mass fixed automatically and detected with an Orbitrap mass analyzer (resolution of 17,500). The dynamic exclusion for the selected ions was 20 s. Maximal ion accumulation time allowed in MS and MS/MS mode were 30 and 50 ms, respectively. Automatic gain control was used to prevent overfilling of the ion trap and was set to 1 × 106 ions and 5 × 104 ions for a full Fourier transform MS and MS/MS scan, respectively.

### 4.17 Protein identification and label free quantitation

The acquired raw MS data files were processed in MaxQuant 1.5.3.30 [18] and searched against the human SwissProt protein database version v 2015.11.11 (42,097 sequences, including isoforms). The search parameters were as follows: two tryptic missed cleavage sites, mass tolerances of 5 ppm and 20 ppm for the precursor and fragment ions, respectively. Oxidation of methionine and N-terminal protein acetylation were set as variable modification, whilst carbamidomethylation of cysteine residues were set as fixed modifications. The data was also matched against a decoy reverse database. Peptides and protein identifications with 1% FDR are reported. Protein identifications requiring a minimum of two peptides sequences were reported. The mass spectrometry proteomics data have been deposited to the ProteomeXchange Consortium via the PRIDE partner repository with the dataset identifier PXD008050.

### 4.18 Bioinformatic analysis

#### 4.18.1 Lipids

The peak intensity was log2-transformed and compared between each condition for timepoints 0 h and 24 h using R version 3.2.2/Bioconductor software package Limma. A linear model was applied for each peak and moderated *t*-tests were computed. In the model, the "condition" was defined as a factor of 3 levels (shame, UVA and UVPAPC). The Benjamini and Hochberg (BH) procedure was applied to adjust the raw p-values into false discovery rate (FDR). A FDR < 0.05 was chosen as the cut-off value. To see the proximities among the conditions in terms of lipids, a Principal Component Analysis was conducted with the conditions in lines and the peaks in columns. Then a heatmap, a double Hierarchical Classification Analysis (HCA) centered and reduced) with Euclidean Distance and the Ward's method, was performed.

### 4.19 Affymetrix array

Robust multi-array average (RMA) signal extraction and normalization were performed using custom chip description file at timepoint 7 h. After exclusion of all Gene IDs with a RMA value of less than 50 in all conditions, log2 transformation was applied. Differential expression between the three conditions (sham, UVA and UVPAPC) was tested using moderated *t*-tests as described above (R version 3.2.2/Bioconductor software package Limma) with a BH multiple testing correction. Gene identifiers covering annotated genes or annotated variants (with eleven probes per set) are referred to as “genes” throughout the manuscript. Genes that were regulated more than 2-fold with a p-value < 0.05 were used for principal component analysis. Heatmaps were used to visualize double HCA of the mRNAs (center and reduced) based on euclidian distance with Ward's method.

### 4.20 Proteins

Protein identifications and LFQ intensities from MaxQuant were analyzed using Perseus statistical package (version 1.5.1.6) [75]. The LFQ intensity values were log2-transformed and zero-intensities were imputed-replaced by normal distribution. Statistical significance of differences in protein levels between groups were evaluated using two-sided *t*-test with p < 0.05 with Permutation based-FDR. PCA and hierarchical clustering analysis using Euclidean distance method for both rows and columns with average linkage and k-mean pre-processing.

### 4.21 Gene network and pathway analyses

Regulated genes and proteins were analyzed with the software QIAGEN's Ingenuity® Pathway Analysis (IPA®, QIAGEN Redwood City, USA, www.qiagen.com/ingenuity) which allowed prediction of activated signaling pathways and upstream regulatory events that were likely to cause the observed gene expression changes, both based on literature evidence. Heatmaps and activation z-scores were calculated within the IPA software package and modified for better presentation as recently described [70].
