# Supplementary material for: A novel role for NUPR1 in the keratinocyte stress response to UV oxidized phospholipids
Source: Redox Biol. 2018 Nov 13;20:467–82. doi: 10.1016/j.redox.2018.11.006 (PMC6243031; doi:10.1016/j.redox.2018.11.006)
Supplement: Supplementary file 2 — Supplementary material [file mmc2.docx]

**S. Table 2a:** List of UVA regulated Gene Identifiers associated with string "lipid" in their gene ontology (GO‐Term)

**Affy. ID Genesymbol ‐log10 adjpval logFC**

130 ABHD4 7,678 1,028

688 AKR1C1 8,358 2,504

689 AKR1C1 8,374 2,529

690 AKR1C1;AKR1C2 7,152 2,043

691 AKR1C2 9,040 2,155

1702 ATP11B 7,916 0,717

1704 ATP11B 8,513 1,051

6955 ELOVL6 8,591 ‐1,033

8078 FLCN 7,007 0,851

8079 FLCN 7,852 1,297

9258 GULP1 7,077 1,620

9259 GULP1 7,626 1,413

9260 GULP1 9,849 1,716

9648 HMGCR 9,357 ‐1,037

9649 HMGCR 7,326 ‐0,947

9650 HMGCR 8,066 ‐0,771

9651 HMGCS1 8,021 ‐0,971

9652 HMGCS1 9,907 ‐1,522

10200 IL1B 10,811 ‐1,758

10335 INSIG1 10,769 ‐2,038

10337 INSIG1 9,379 ‐1,582

10439 IRS2 7,476 1,389

11429 LDLR 9,379 ‐1,432

11430 LDLR 8,393 ‐1,171

11835 LRP8 8,109 ‐1,081

11836 LRP8 8,352 ‐1,306

12600 MEST 10,196 ‐1,162

12601 MEST 7,916 ‐1,122

14758 OSBP 7,272 1,082

14759 OSBP 8,130 1,100

14772 OSBPL2 7,630 0,821

15956 PNPLA8 7,491 1,373

15958 PNPLA8 9,891 1,410

16849 PTGS2 8,295 1,497

16850 PTGS2 8,453 1,579

26782 PTGS2 7,249 1,925

18615 SC4MOL 7,407 ‐1,122

18616 SC4MOL 8,543 ‐1,333

18617 SC4MOL 8,693 ‐0,879

20616 ST3GAL5 10,543 ‐1,315

**S. Table 2b:** List of UVPAPC regulated Gene Identifiers associated with string "lipid" in their gene ontology (GO‐Term)

**Affy. ID Genesymbol ‐log10 adjpval logFC**

130 ABHD4 8,363 1,267

688 AKR1C1 9,895 3,899

689 AKR1C1 9,895 3,915

690 AKR1C1;AKR1C2 8,363 2,850

691 AKR1C2 10,163 3,074

27648 AKR1C2 6,380 1,770

1702 ATP11B 8,448 0,862

1704 ATP11B 8,144 1,008

7247 ETNK1 6,720 1,157

8950 GPAM 8,299 ‐1,509

8951 GPAM 8,125 ‐1,651

9260 GULP1 7,476 1,003

10200 IL1B 9,237 ‐1,211

11773 LPCAT1 9,296 ‐1,293

15958 PNPLA8 8,363 1,013

16849 PTGS2 8,399 1,615

16850 PTGS2 8,762 1,810

26782 PTGS2 7,304 2,028
