# Supplementary material for: A novel role for NUPR1 in the keratinocyte stress response to UV oxidized phospholipids
Source: Redox Biol. 2018 Nov 13;20:467–82. doi: 10.1016/j.redox.2018.11.006 (PMC6243031; doi:10.1016/j.redox.2018.11.006)
Supplement: Supplementary file 5 — Supplementary material [file mmc5.docx]

AGPAT2 2,204 ‐0,918

EDF1 1,876 0,667

HMGCS1 2,492 ‐0,637

IDI1 3,072 0,652

OSBP 7,056 2,316

PLD1 1,965 0,781

RFT1 1,784 ‐1,639

SGPL1 4,870 0,657

TECR 4,940 0,607

ACACA 1,413 0,696

ACLY 2,912 0,666

ACOX1 2,052 ‐1,664

ACSL4 7,387 ‐1,102

AKR1C1 1,772 1,915

ANXA1 3,399 0,603

EHHADH 2,045 ‐0,804

FABP5 2,672 0,801

FADS2 10,207 ‐3,494

FASN 4,101 0,809

FDFT1 2,269 ‐1,098

GM2A 1,810 0,853

GPX1 2,980 0,756

HADHB 2,118 0,600

HMGCR 7,577 ‐3,859

HMGCS1 4,978 ‐1,260

IDH1 3,994 0,672

LDLR 3,932 0,791

NFKB1 2,764 1,119

OSBP 2,312 1,985

PAFAH1B3 1,410 1,130

PCNA 5,894 ‐0,857

PRDX6 3,213 1,145

PRKAA1 1,893 ‐1,297

PSAP 3,213 0,901

PTDSS1 1,810 ‐1,413

PTGES3 1,963 2,049

PTGS2 2,639 0,865

SCD 3,911 ‐1,592

SOAT1 2,579 ‐1,294

TPI1 3,288 0,668

TPP1 6,861 0,640

UGCG 2,935 ‐2,639

VPS4B 2,249 1,617
