# Supplementary material for: A novel role for NUPR1 in the keratinocyte stress response to UV oxidized phospholipids
Source: Redox Biol. 2018 Nov 13;20:467–82. doi: 10.1016/j.redox.2018.11.006 (PMC6243031; doi:10.1016/j.redox.2018.11.006)
Supplement: Supplementary file 7 — Supplementary material [file mmc7.docx]

**Supplementary Material**

**A novel role for NUPR1 in the keratinocyte stress response to UV oxidized phospholipids.**

Marie-Sophie Narzt^1,2^, Ionela-Mariana Nagelreiter^1,2^, Olga Oskolkova^3^; Valery N. Bochkov^3^ , Julie Latreille^4^, Maria Fedorova^5,6^, Zhixu Ni^5,6^, Fernando J. Sialana^7^, Gert Lubec^8^, Manuel Filzwieser²,Maria Laggner^9^, Martin Bilban^10^, Michael Mildner^1^, Erwin Tschachler^1^, Johannes Grillari^2,11^, Florian Gruber^1,2^

*^1^ Department of Dermatology, Medical University of Vienna, Vienna, Austria. ^2^Christian Doppler Laboratory for Biotechnology of Skin Aging, Austria. ^3^ Institute of Pharmaceutical Sciences, University of Graz, Graz, Austria. ^4^ Department of Biology & Women’s Beauty, Chanel, Pantin, France. ^5^ Institute of Bioanalytical Chemistry, Faculty of Chemistry and ^6^Center for Biotechnology and Biomedicine, Universität Leipzig, Leipzig, Germany. ^7^Department of Pharmaceutical Chemistry, Faculty of Life Sciences, University of Vienna, Vienna, Austria. ^8^ Paracelsus Medical University of Salzburg, Salzburg, Austria. ^9^ Department of Ophthalmology and Optometry, Medical University of Vienna,Vienna Austria*, *^10^ Department of Laboratory Medicine & Core Facility Genomics, Medical University of Vienna, Vienna, Austria. ^11^Department of Biotechnology, BOKU, University of Natural Resources and Life Sciences Vienna, Austria.*

**Supplementary Figure 1**

**
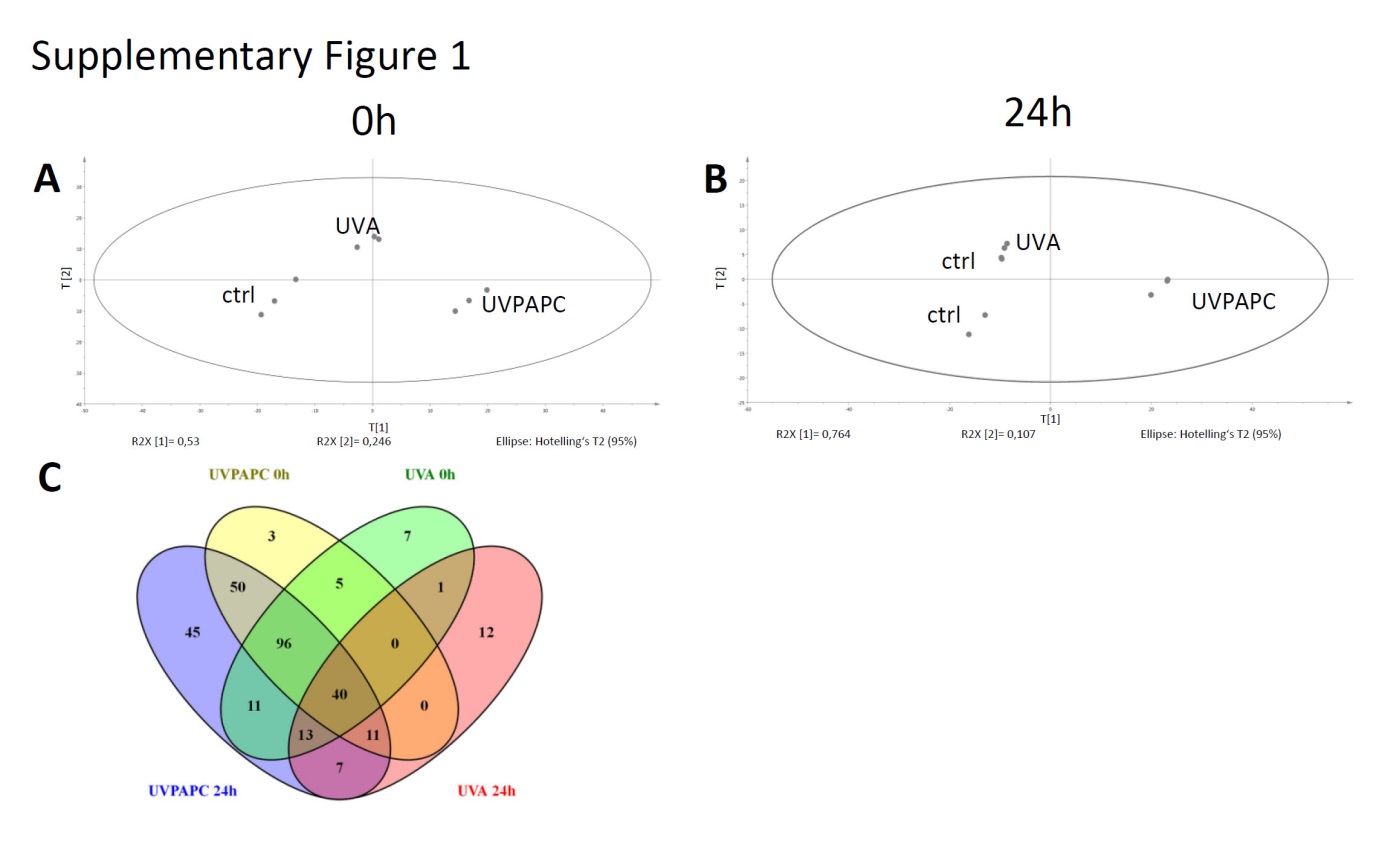
**

**Supplementary Figure 1 - Principal component analysis and Venn diagram of the oxidized phosphocholine profile of human KC.** Principal component analysis (PCA) of the regulated oxidized phospholipids was performed with all samples and is displayed in panel **A** for the oxidized PCs harvested immediately after stress and in panel **B** 24h post stress treatment. **C** Venn diagram showing the overlap of the oxidized PC species of the three conditions (sham, UVA and UVPAPC) and the two time points (0h and 24h).


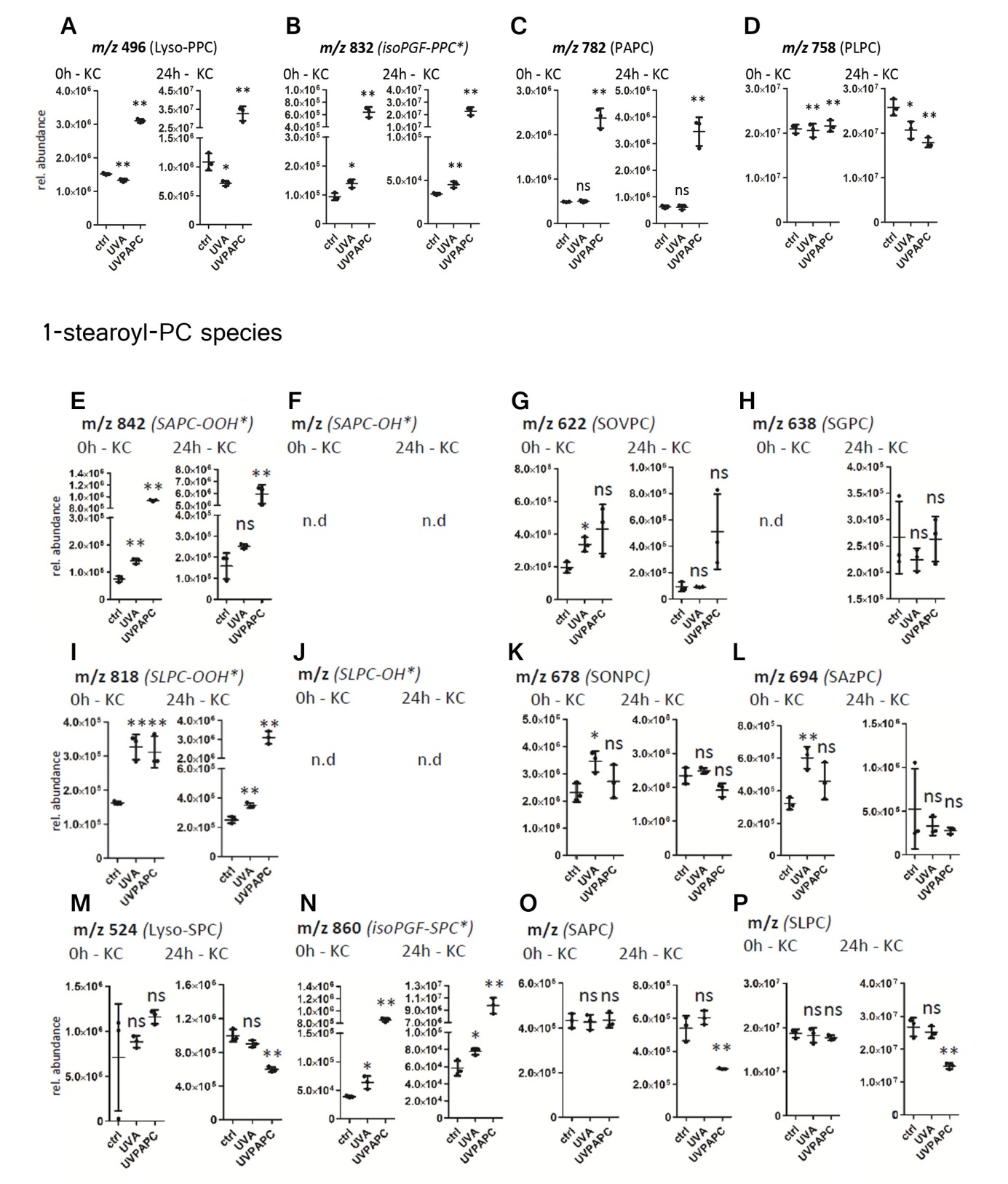


**Supplementary Figure 2 and lipidomic analysis of additional PPC species and *sn*-1 stearoyl PC (SPC) species**

Dot blots showing relative abundance of **A** Lyso-PPC, **B** isoPGF-PPC, **C** Un-oxidized PAPC and **D** un-oxidized PLPC; **E-P** Dot blots showing relative abundance of the respective 1-steaoryl-PC species 0h and 24h post stress treatment. (n=3; normalized to DPPC; error bars indicate SD). Asterisks indicate significant differences (*P<0.05; ** P<0.01) determined by Student’s t-test.

**Text Supplementary Figure2** In Supplementary Figure 2 **E-P** the corresponding species with stearic instead of palmitic acid in the *sn*-1 position of the PL are shown. Lysophospholipid levels were slightly decreased upon UVA exposure (Fig. S2 **A,M**). Species at *m/z* 832 and 860 (Fig. S2 **B,N**), earlier reported as isoprostanoid modifications of the arachidonic acid moieties of PAPC and SAPC, respectively, were elevated upon both treatments, strongest upon UVPAPC. The unoxidized precursor lipid levels (Fig. S2 **C,D**) were unchanged, except for PAPC in those samples that had been treated with UVPAPC, which contains a high percentage of native PAPC. UVPAPC treatment decreased the DPPC - normalized levels of PLPC, SAPC and SLPC after 24 hours (Fig S2 **D,O,P**).

**Supplementary Figure 3 - Analysis strategy for un-identified regulated PL species using high resolution tandem MS**

The structures for *m/z* 596, 550, 664, 546 and 800 shown in Fig. 3 and in subpanels **A1,B1,C1,D1,E1** of this figure were proposed based on combining information from positive (subpanels **A2 -E2**) and negative (subpanels **A4-E4**) ion mode exact mass extracted ion chromatograms (XIC, 20 ppm mass tolerance) with collision induced dissociation (CID) analysis in the positive mode to confirm the PC diagnostic fragment at *m/z* 184.1 (subpanels **A3-E3**). Negative ion mode tandem mass spectra identify the fatty acid composition (subpanels **A5-E5**).

In detail, candidate signals identified with the screening lipidomic method (Fig. 2) were in-depth re-analyzed with high resolution MS following the same strategy in subpanels A-E. First, in the positive ion mode, the XIC for the signal at a given *m/z* identified in the screening experiment *(*596, 550, 664, 546 and 800) were analyzed within a 0.3 Da range to identify all possible signals, and accurate m/z were determined. Next, an XIC for each of these signals was prepared within 20ppm mass tolerance (A2-E2), and HPLC retention times were determined. For each peak the monoisotopic signal at the correct *m/z* value was confirmed. Then the MS/MS scan (product ion at m/z 184 for PC headgroup) confirms the signals as PC lipids (A3-E3). Next, the presence of the signal in the negative ion mode and it’s monoisotopic signal at the correct m/z value were confirmed. The corresponding precursor deprotonated formate adducts [M+H_2_CO_2_-H]^-^ (A4-E4) were used to assign the fatty acid composition (A5-E5) and propose the structures.


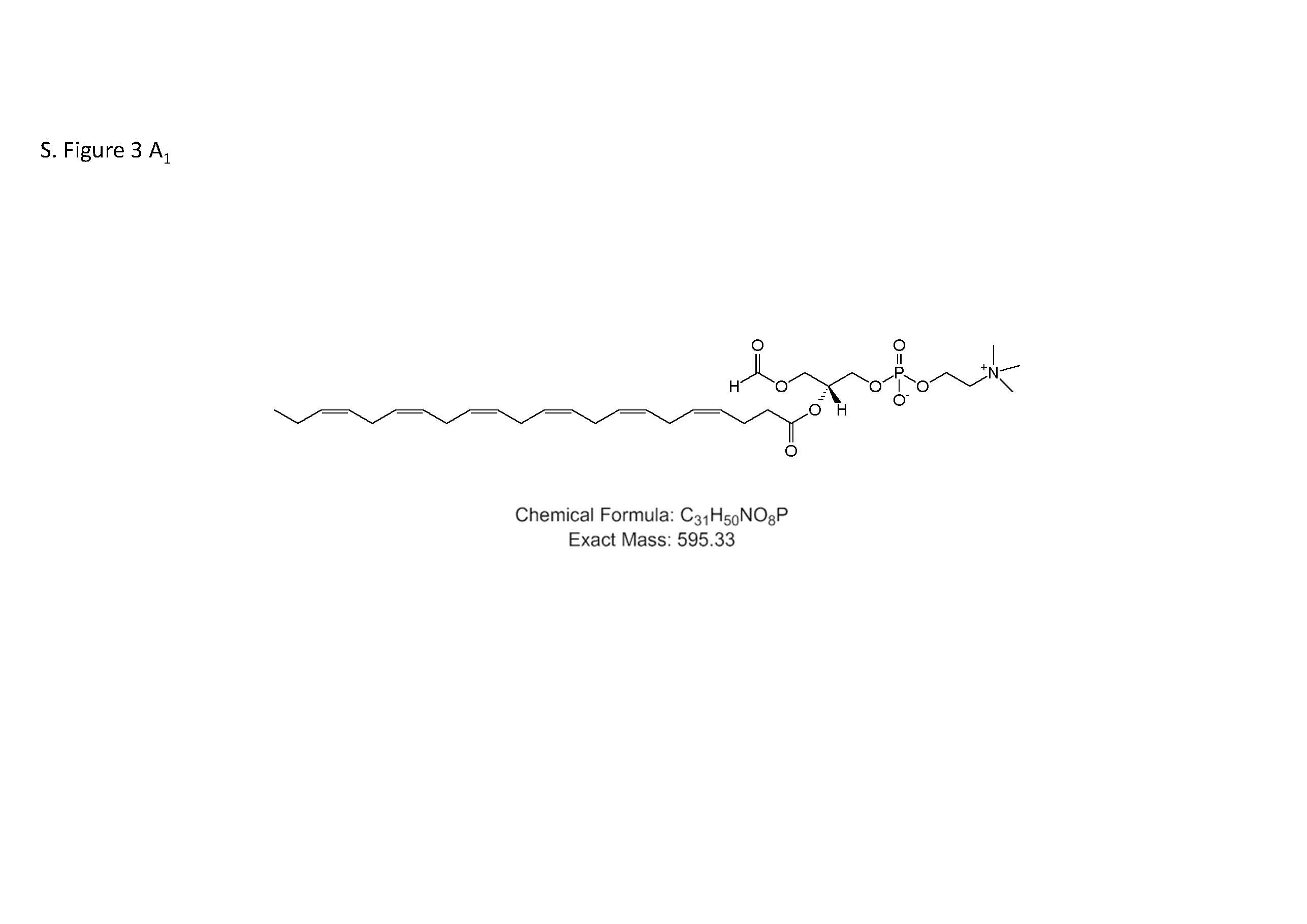


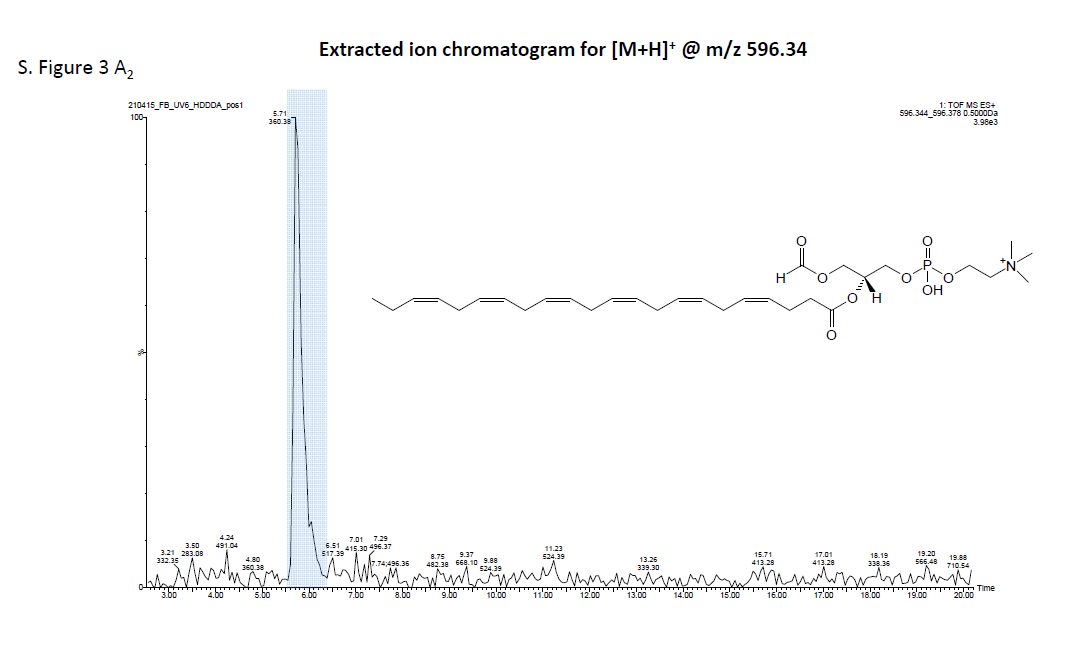


**
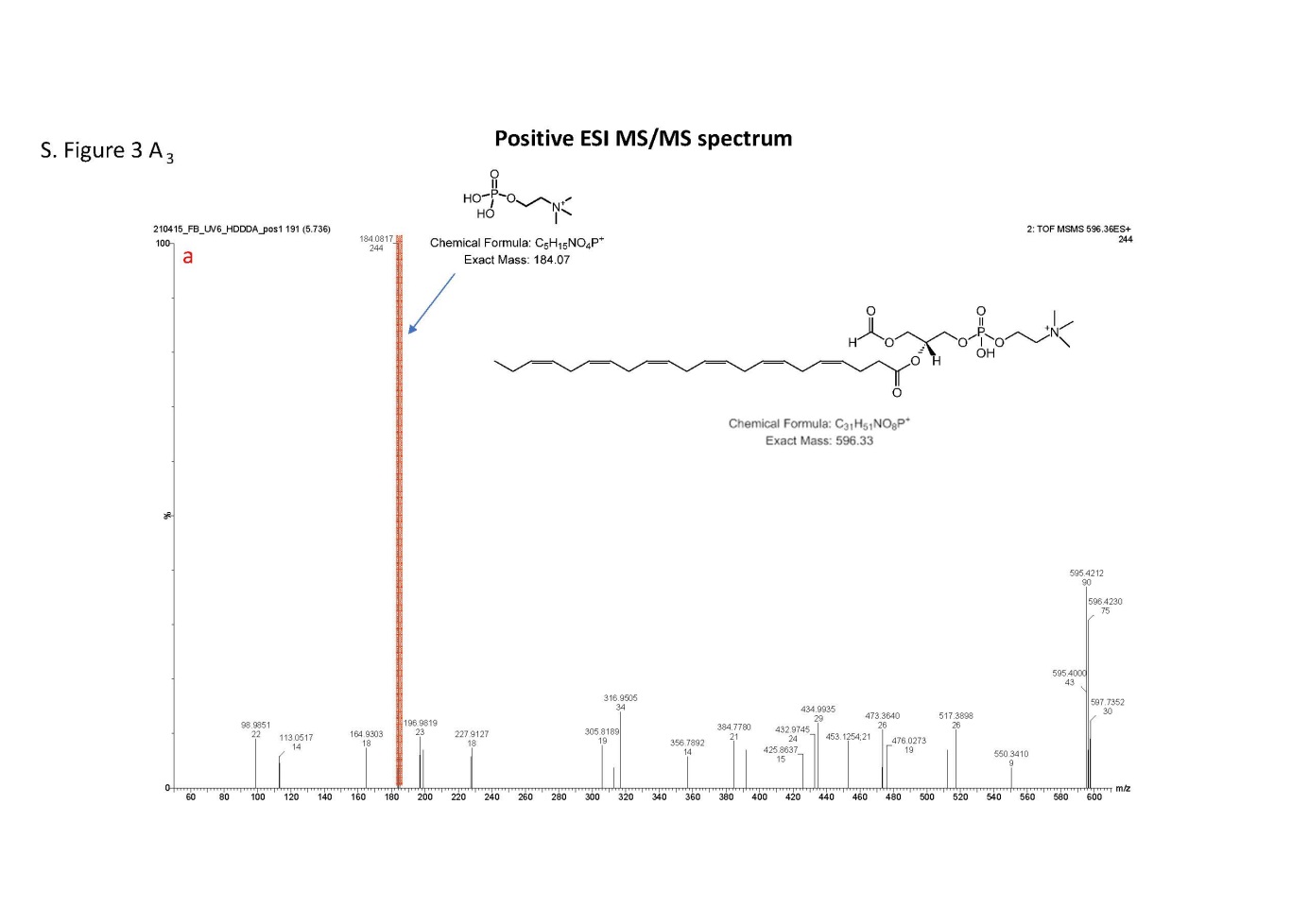
**

**
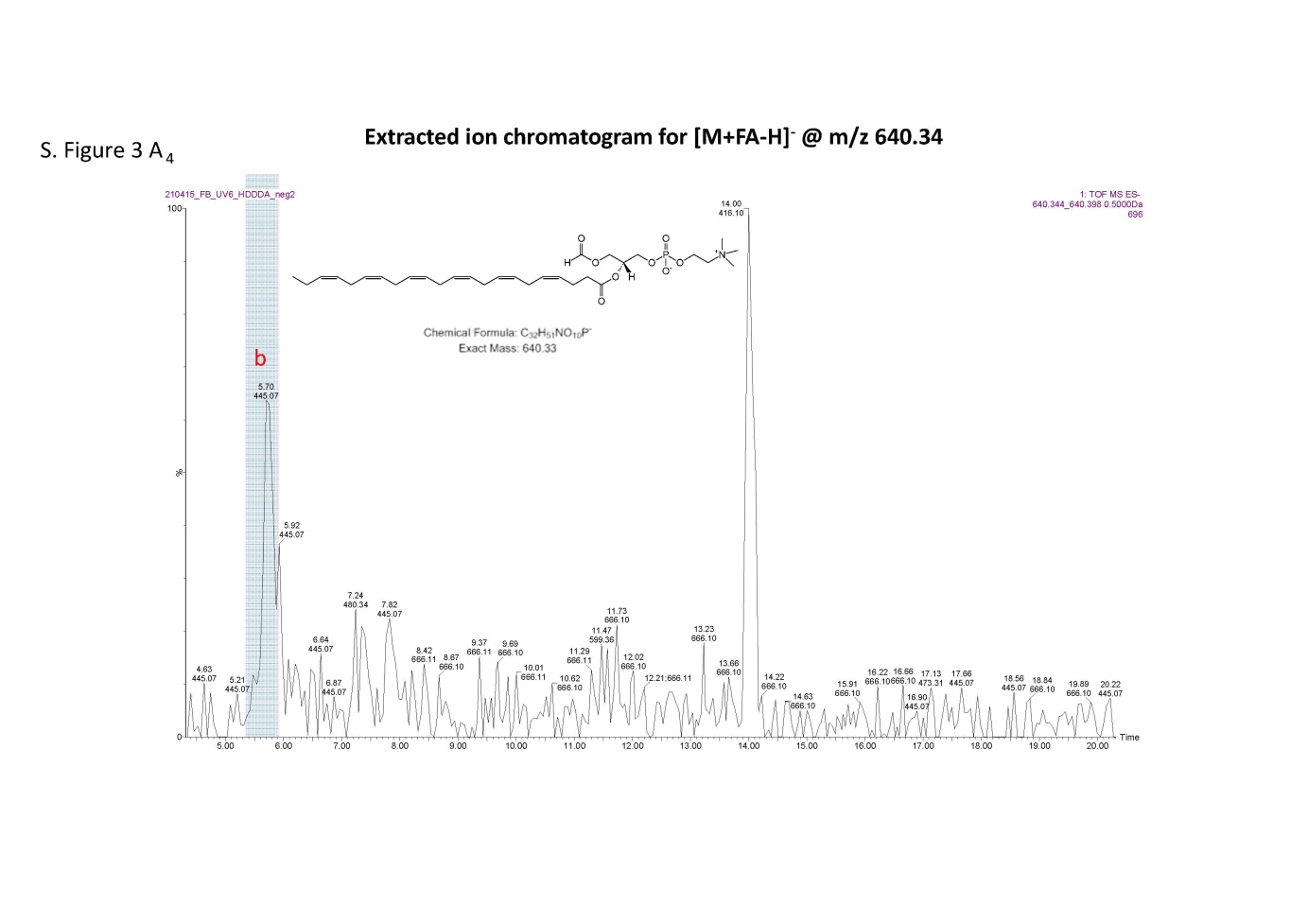
**

**
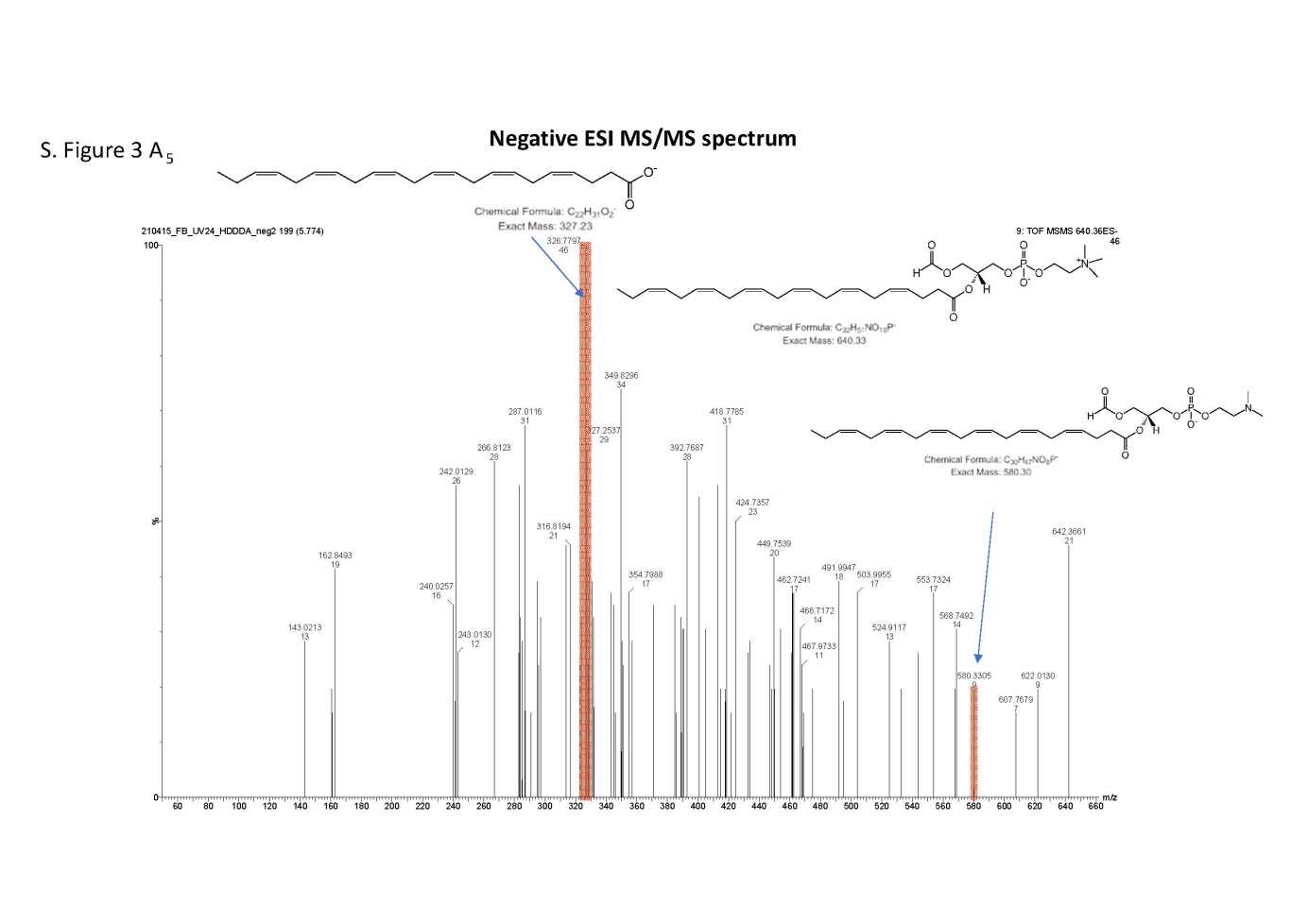

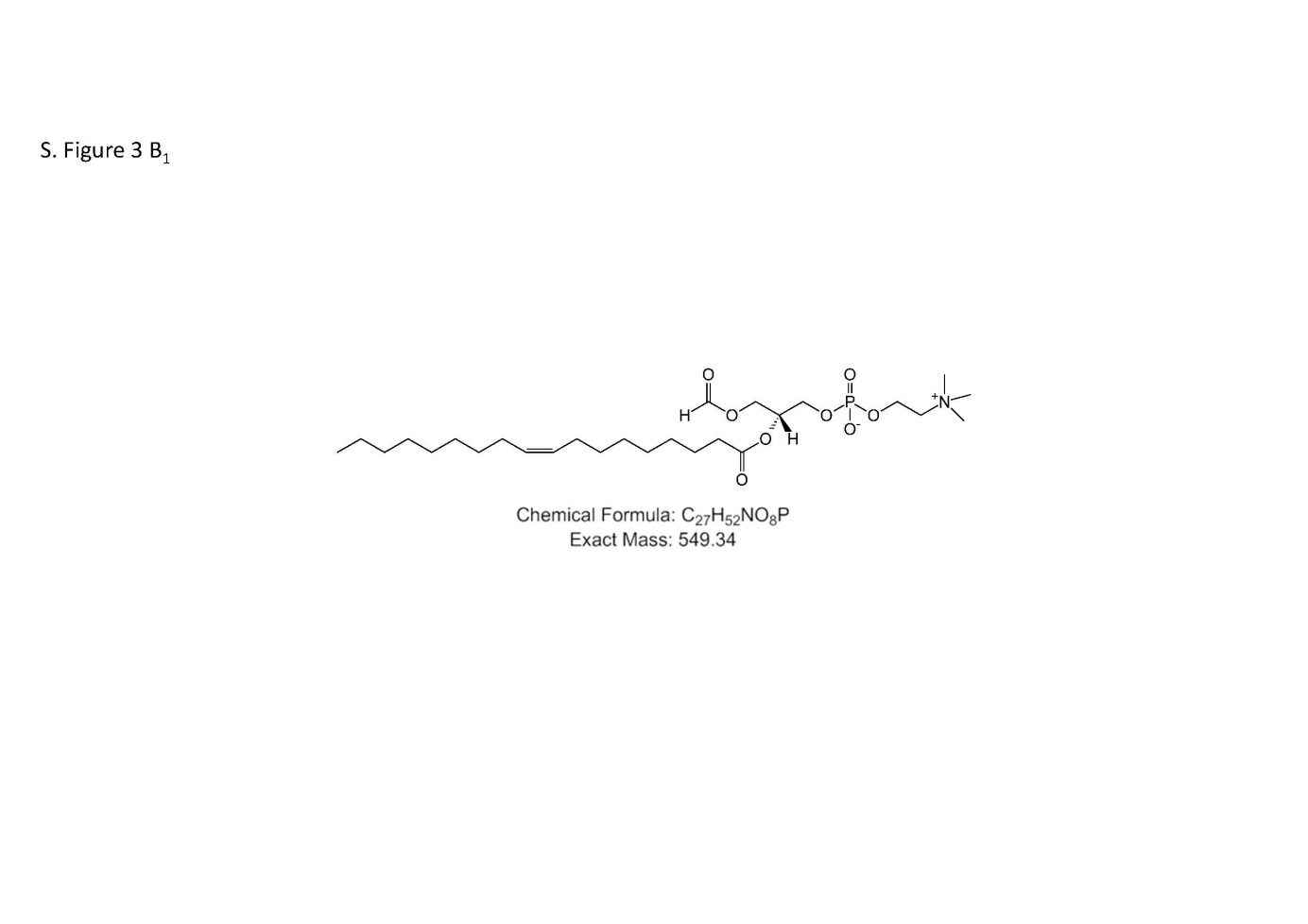

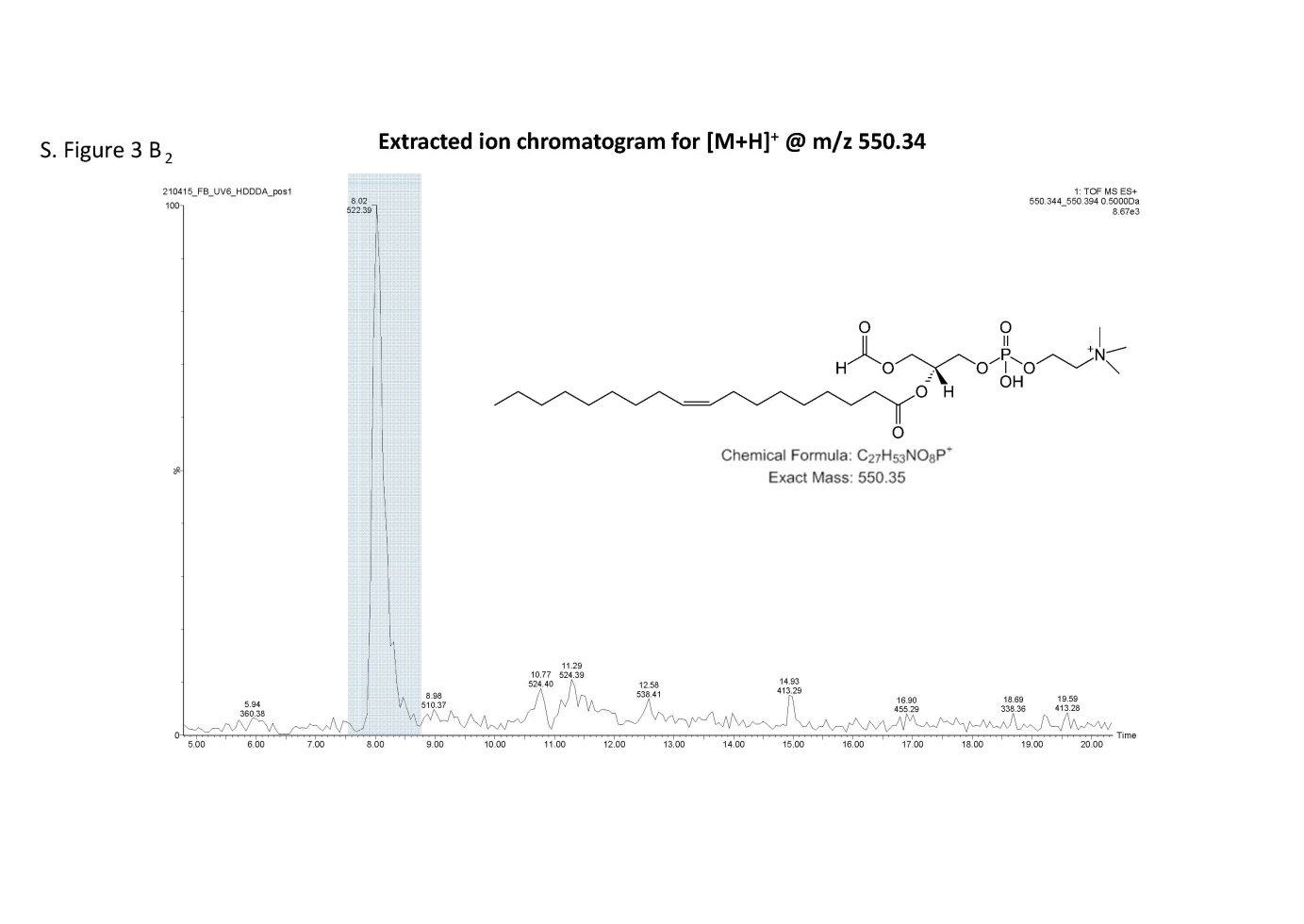

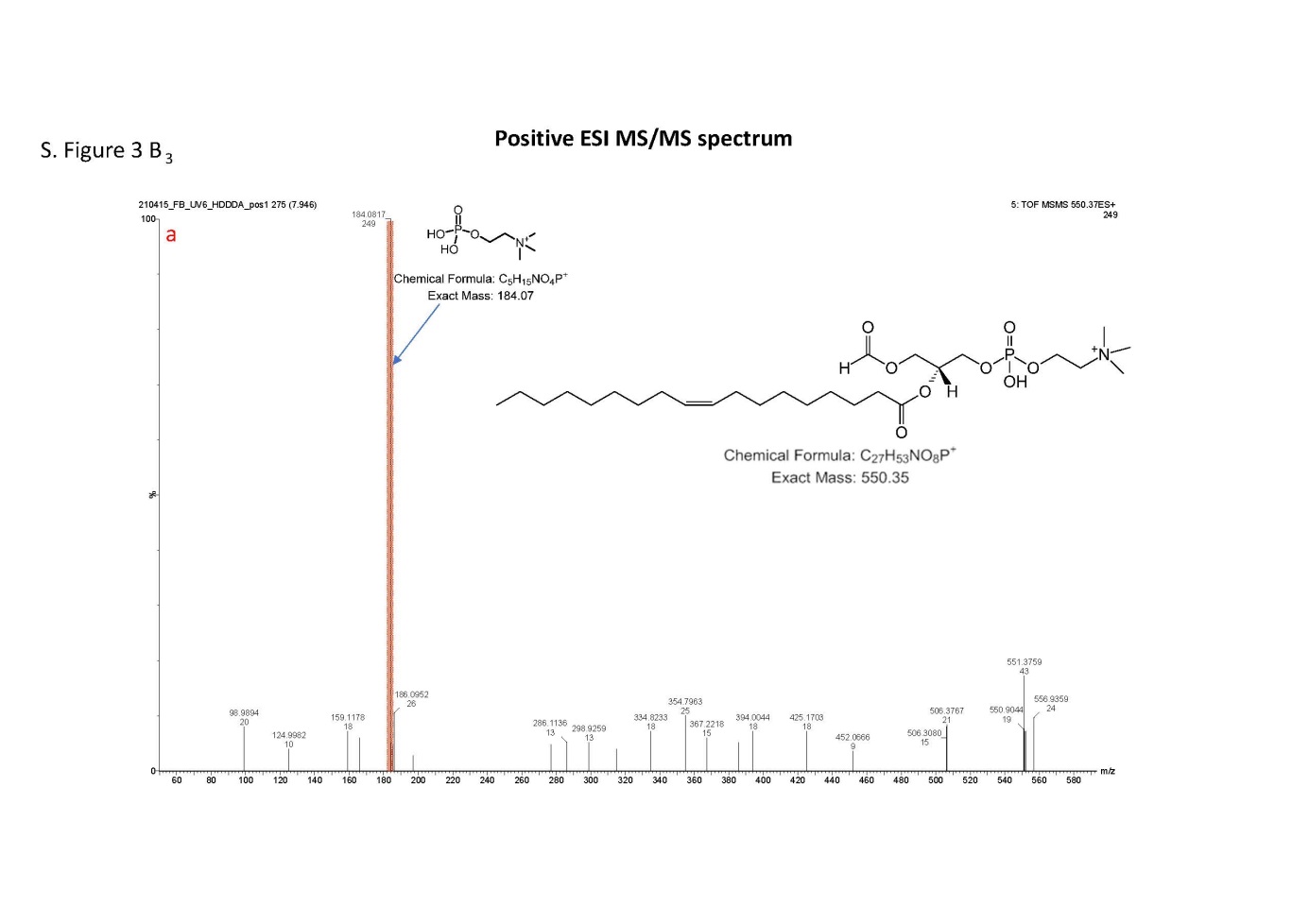

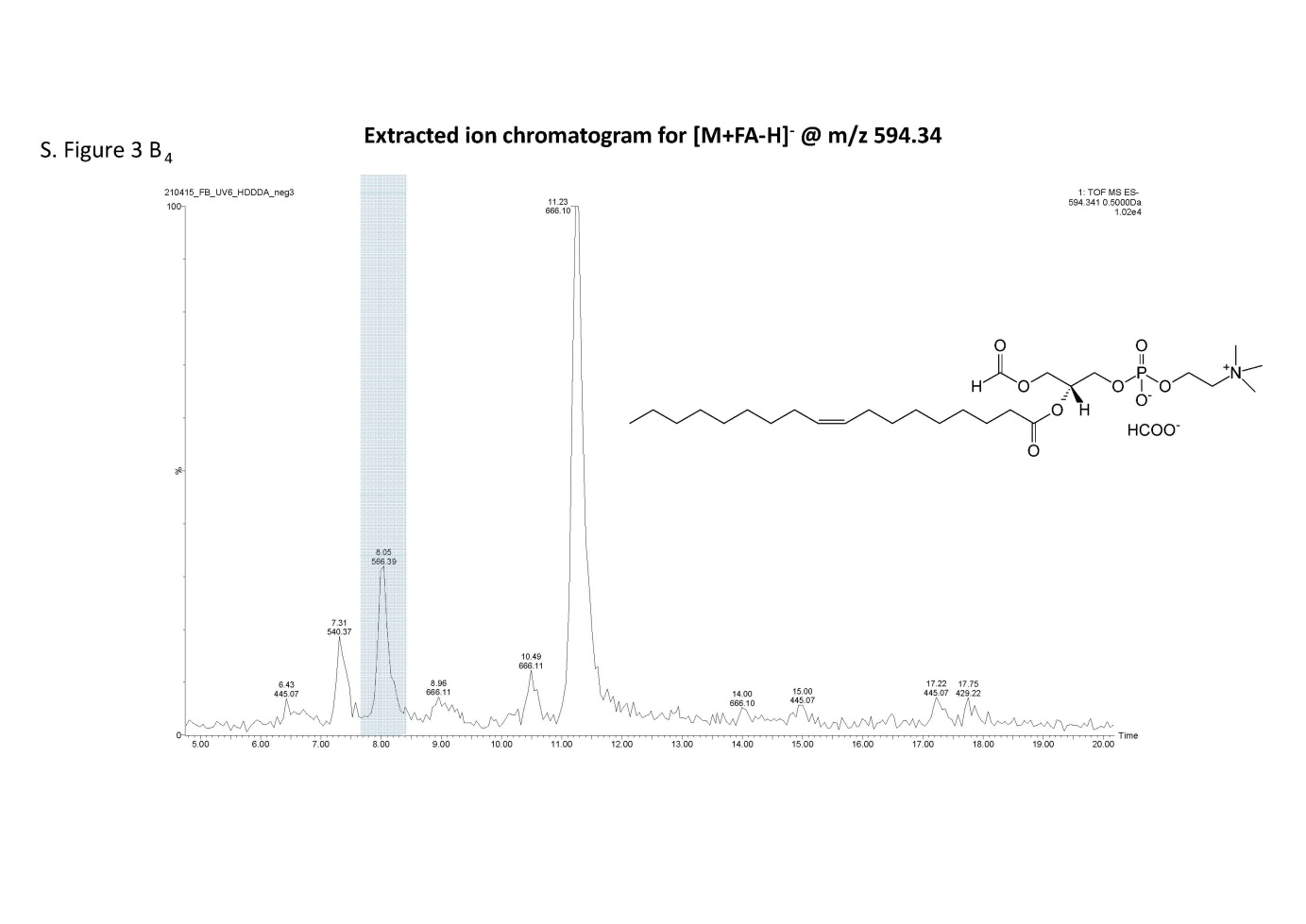

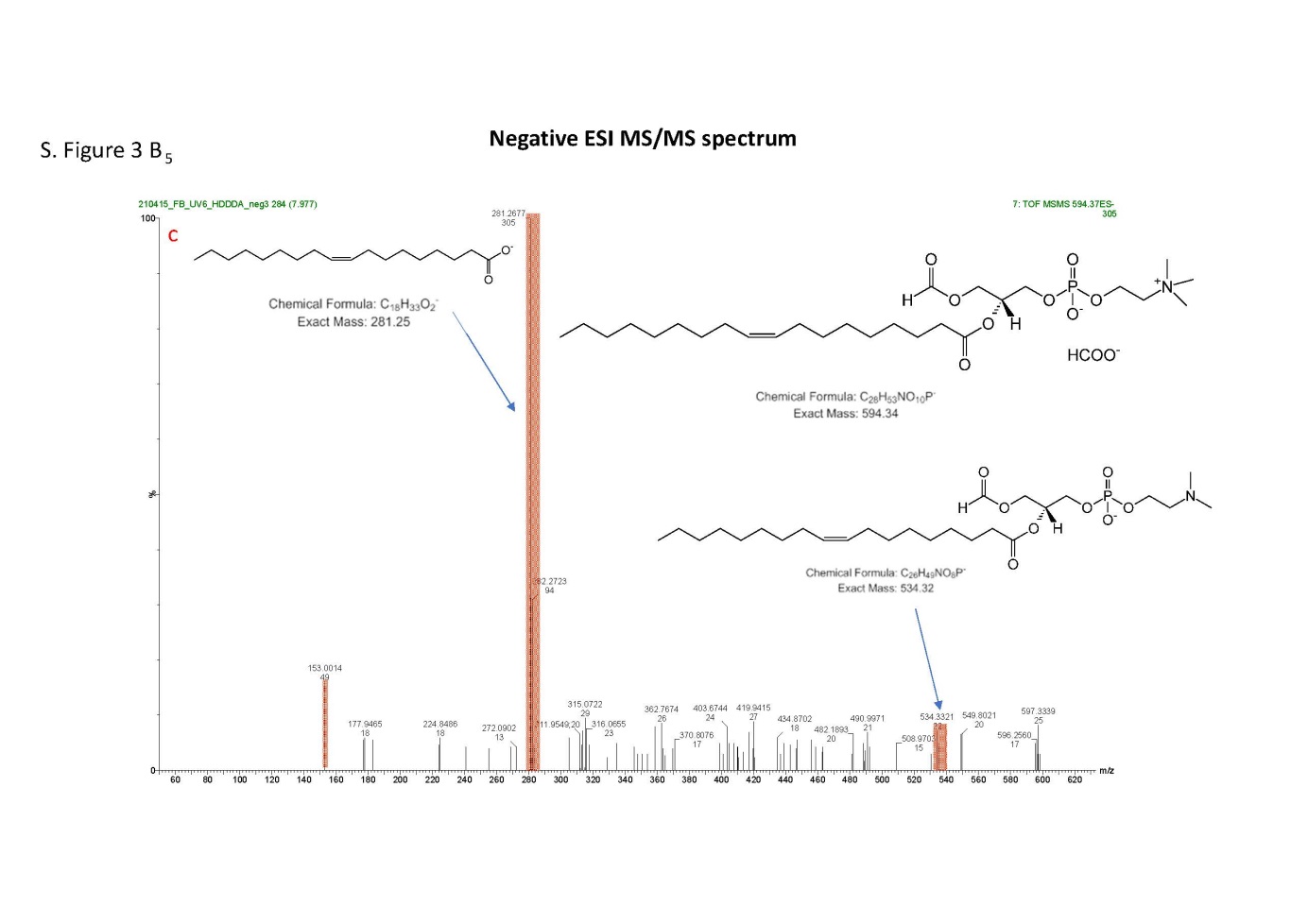
**


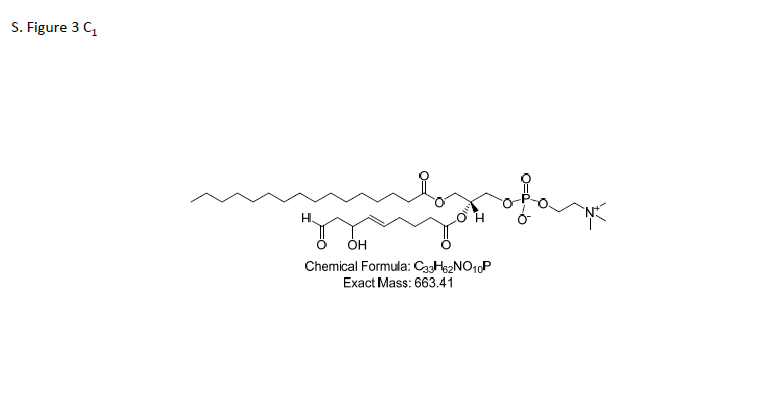


**
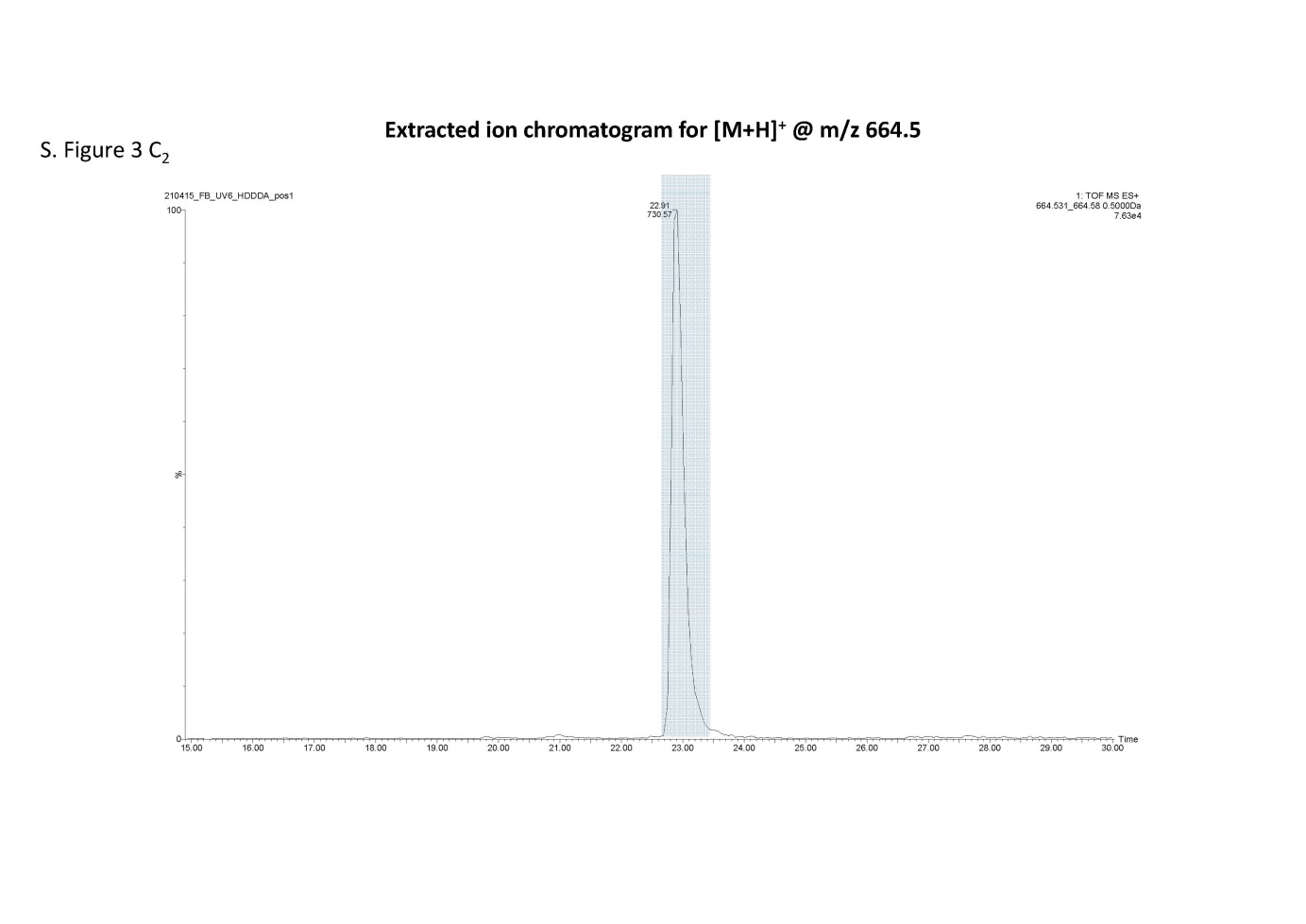
**

**
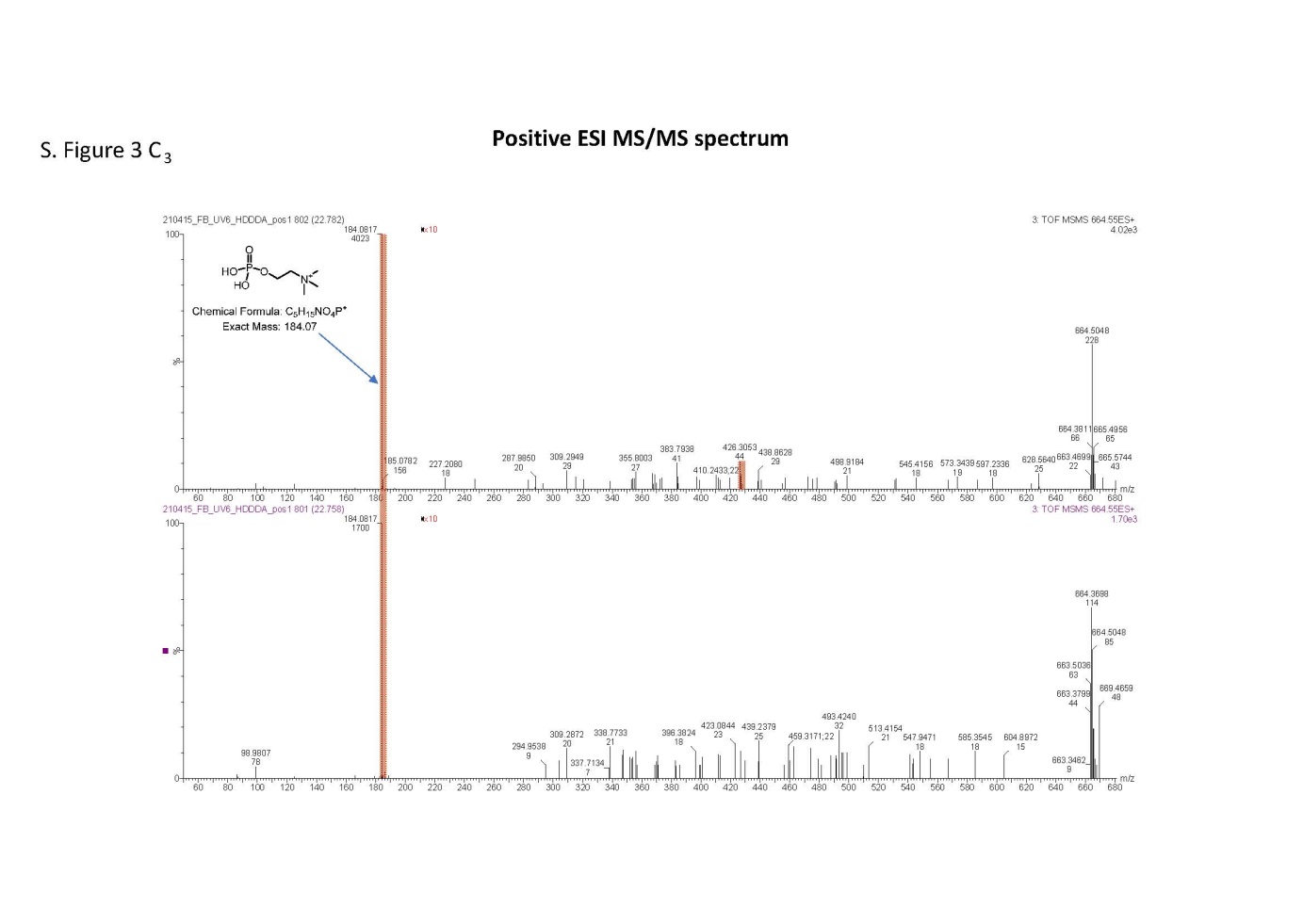

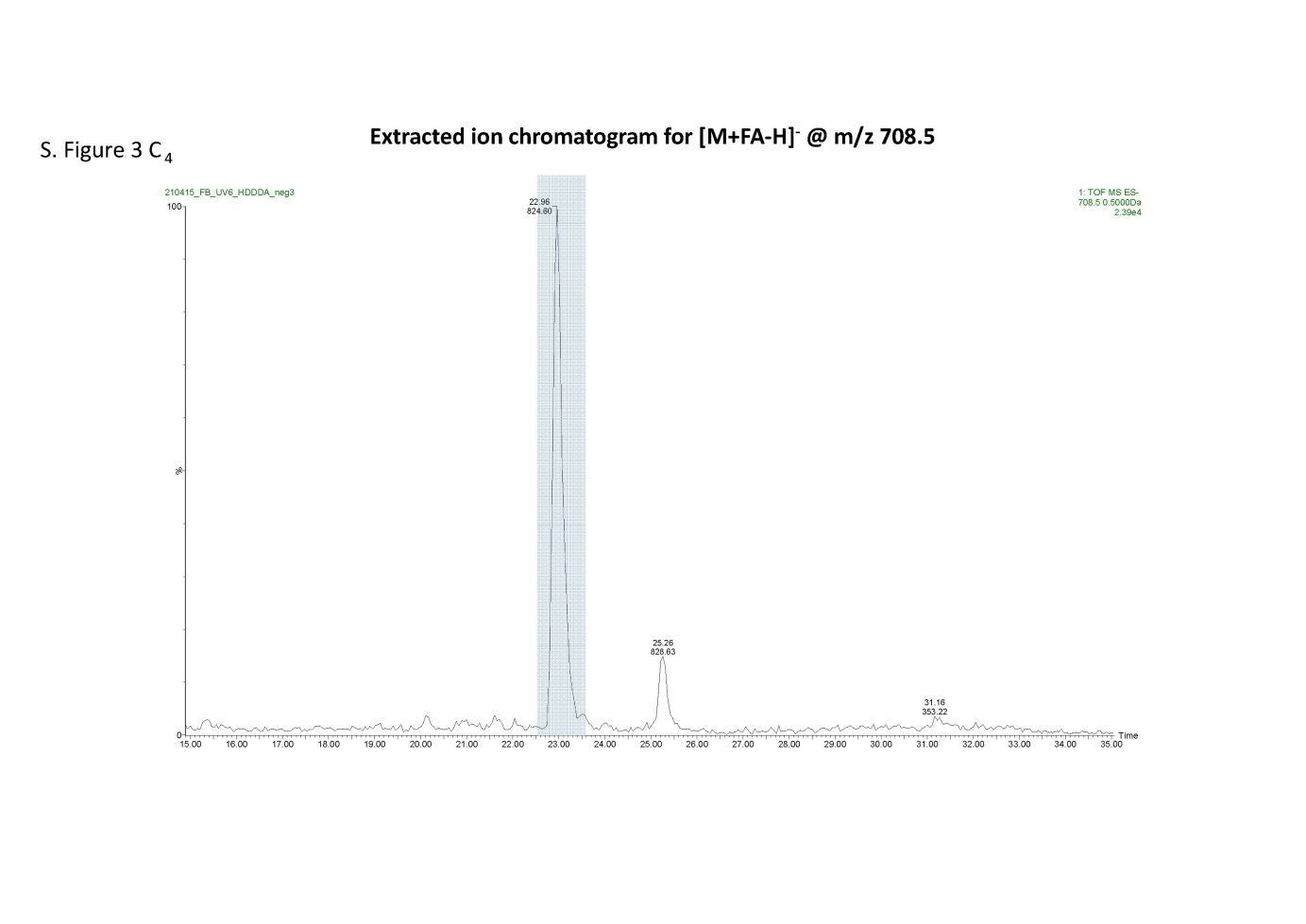

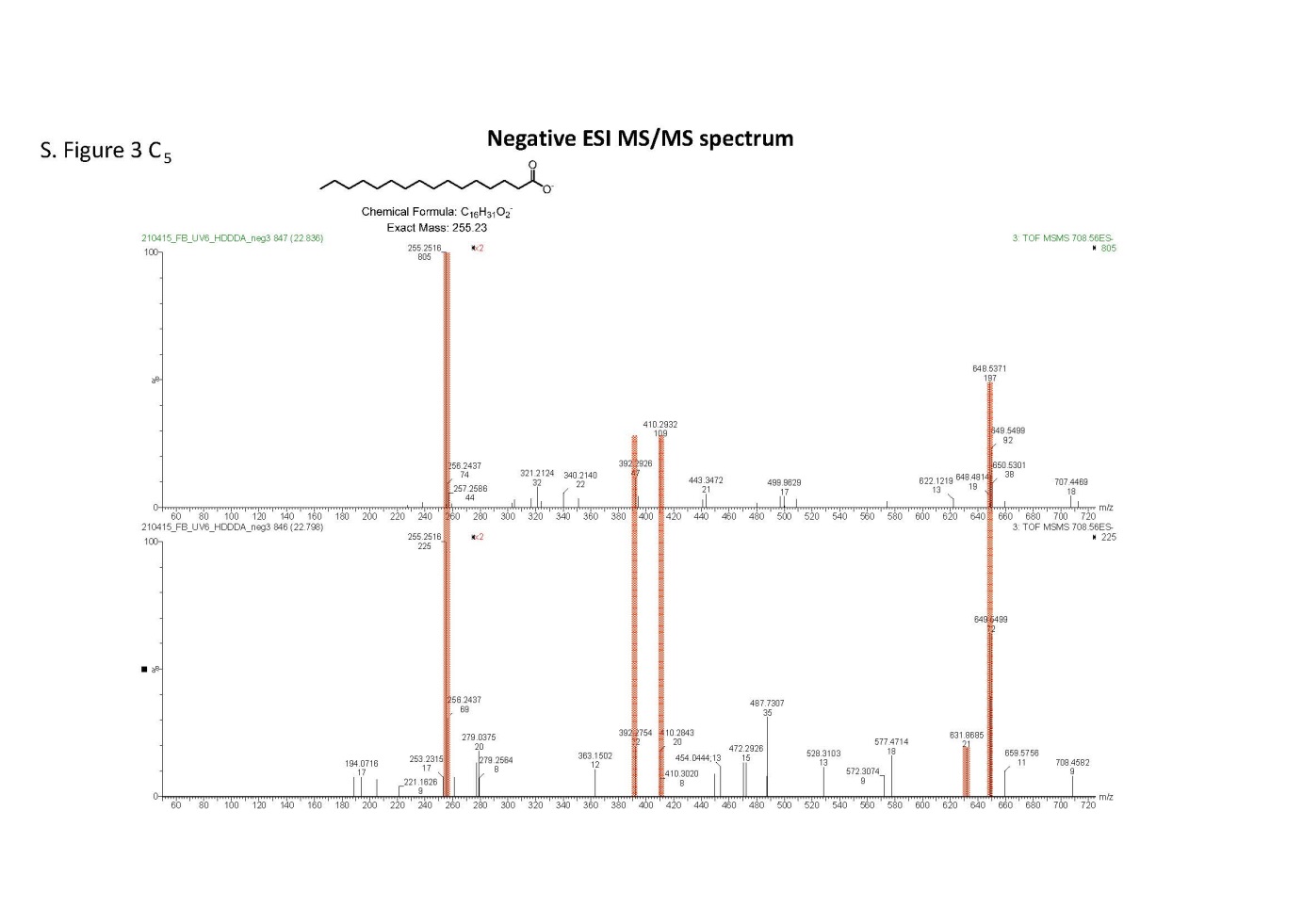
**


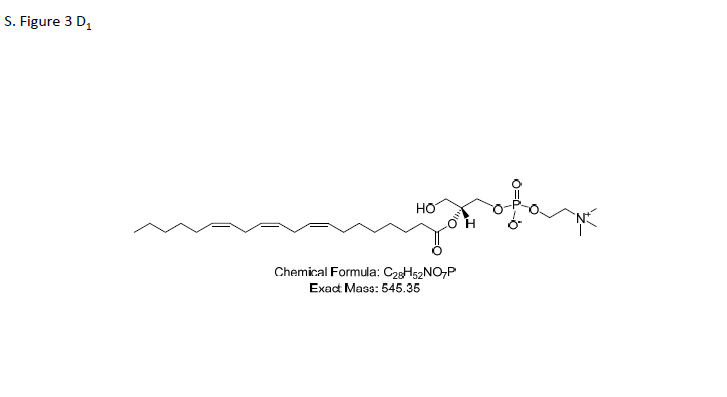
**
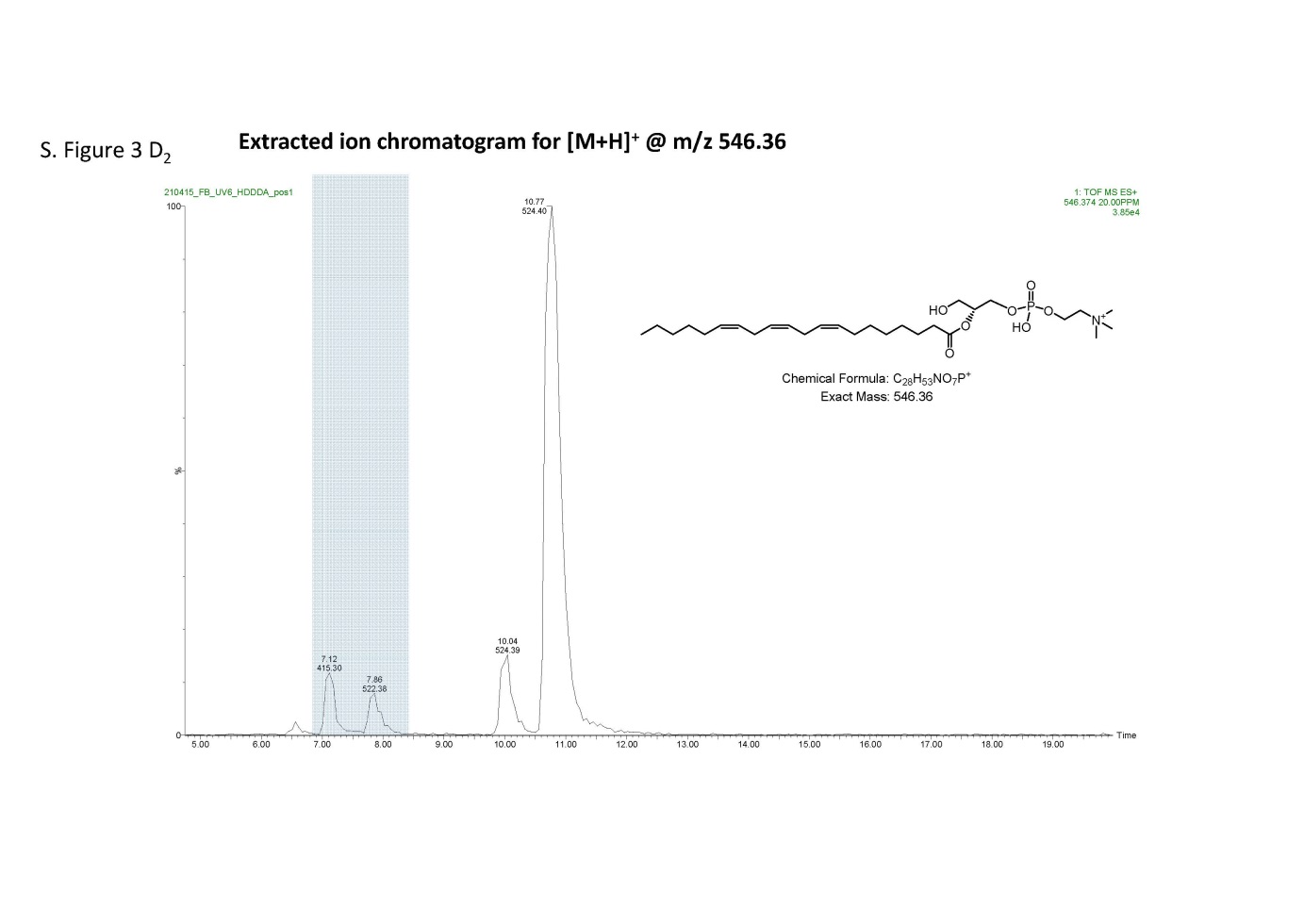
**

**
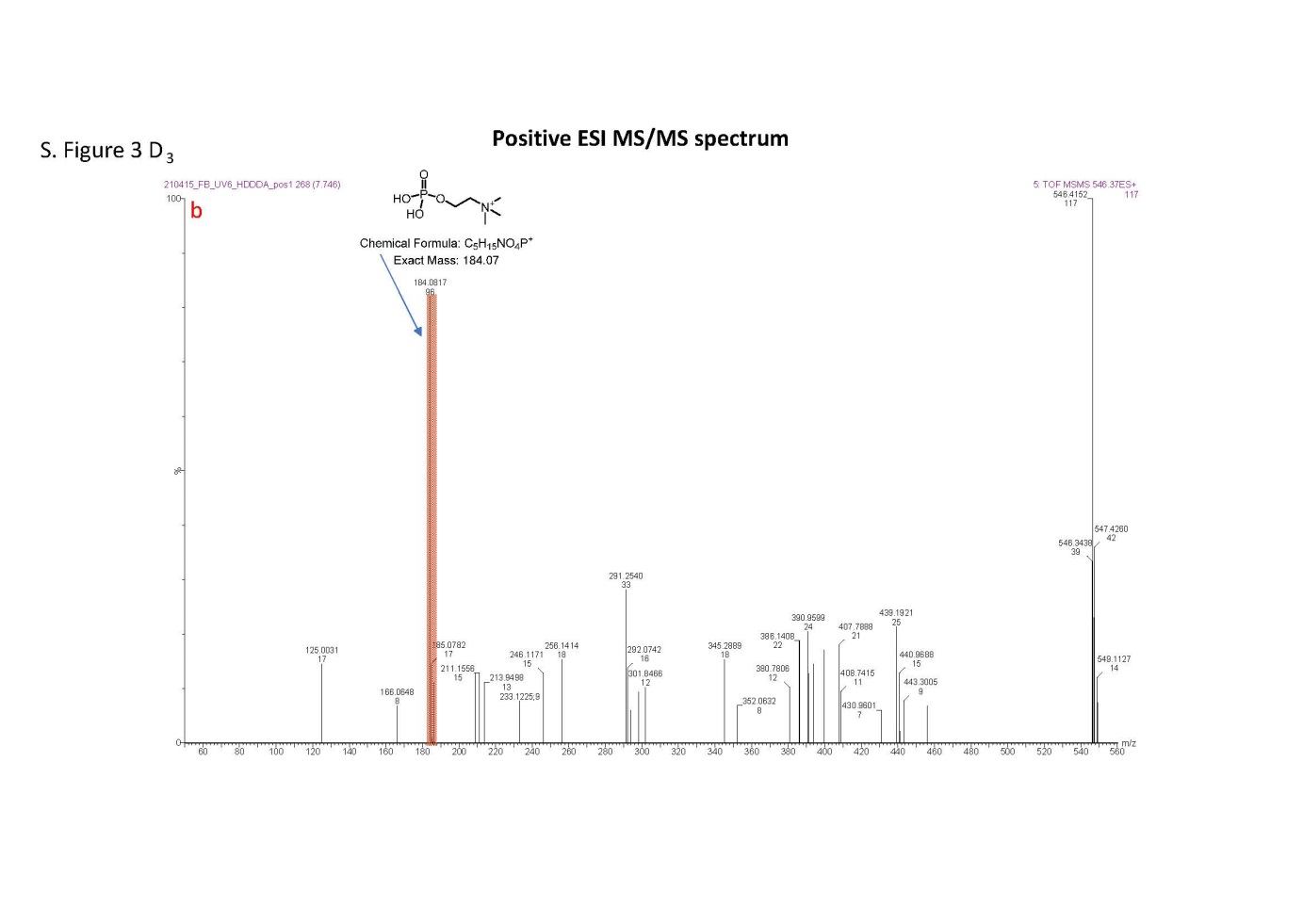
**

**
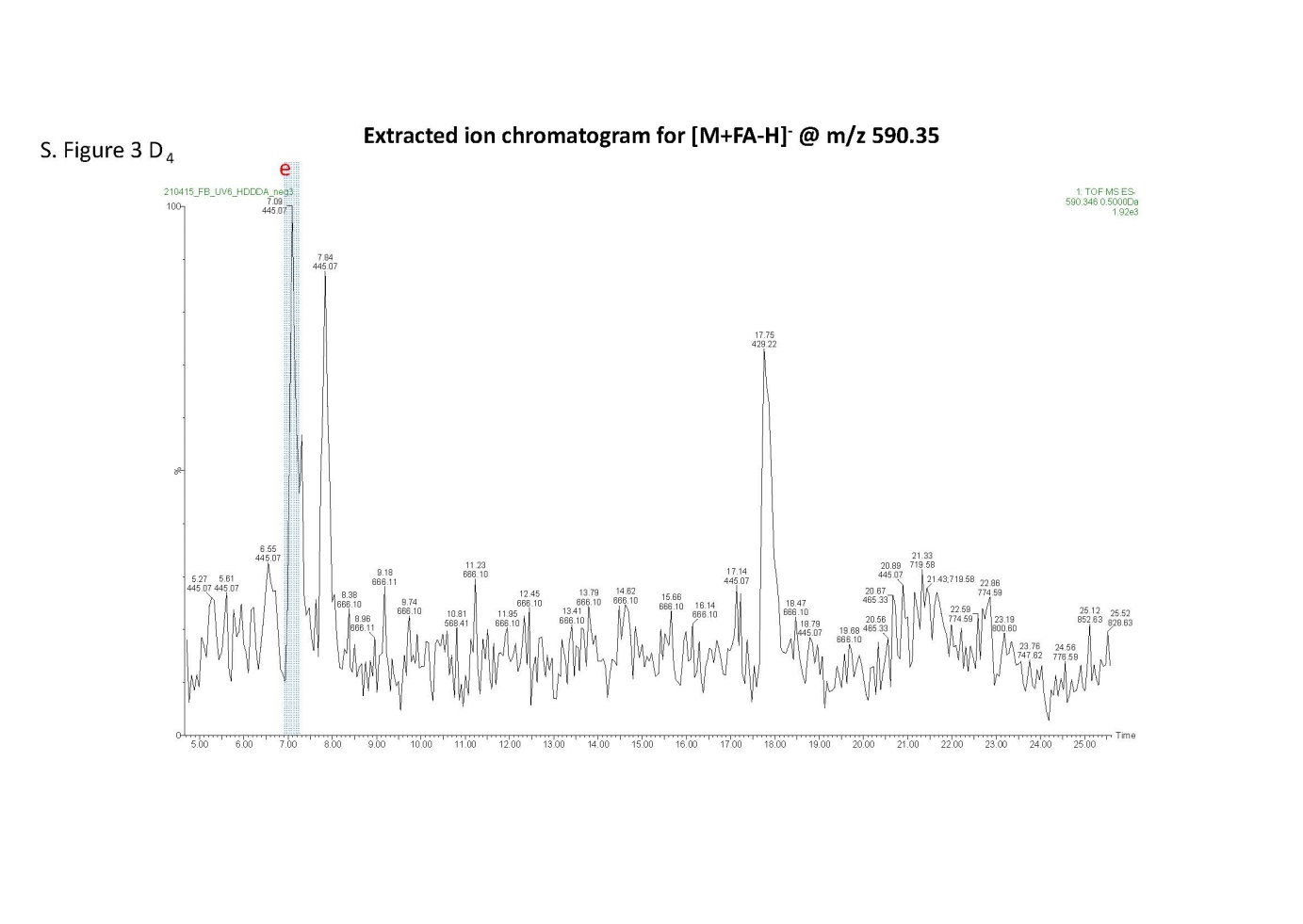
**


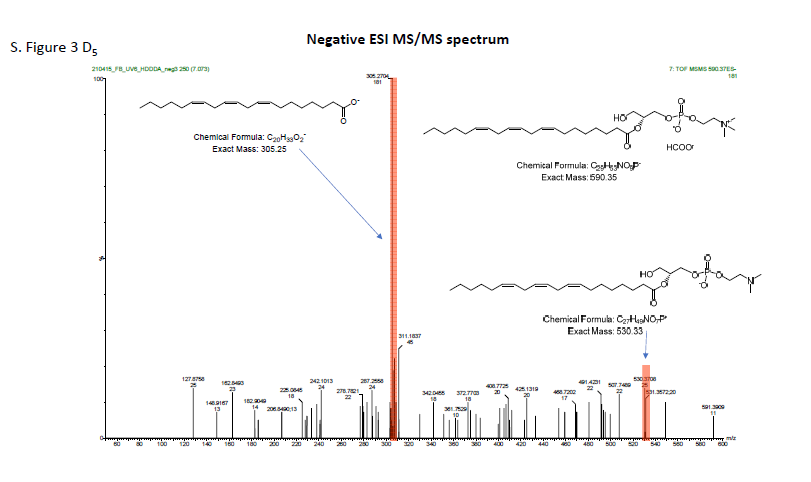


**
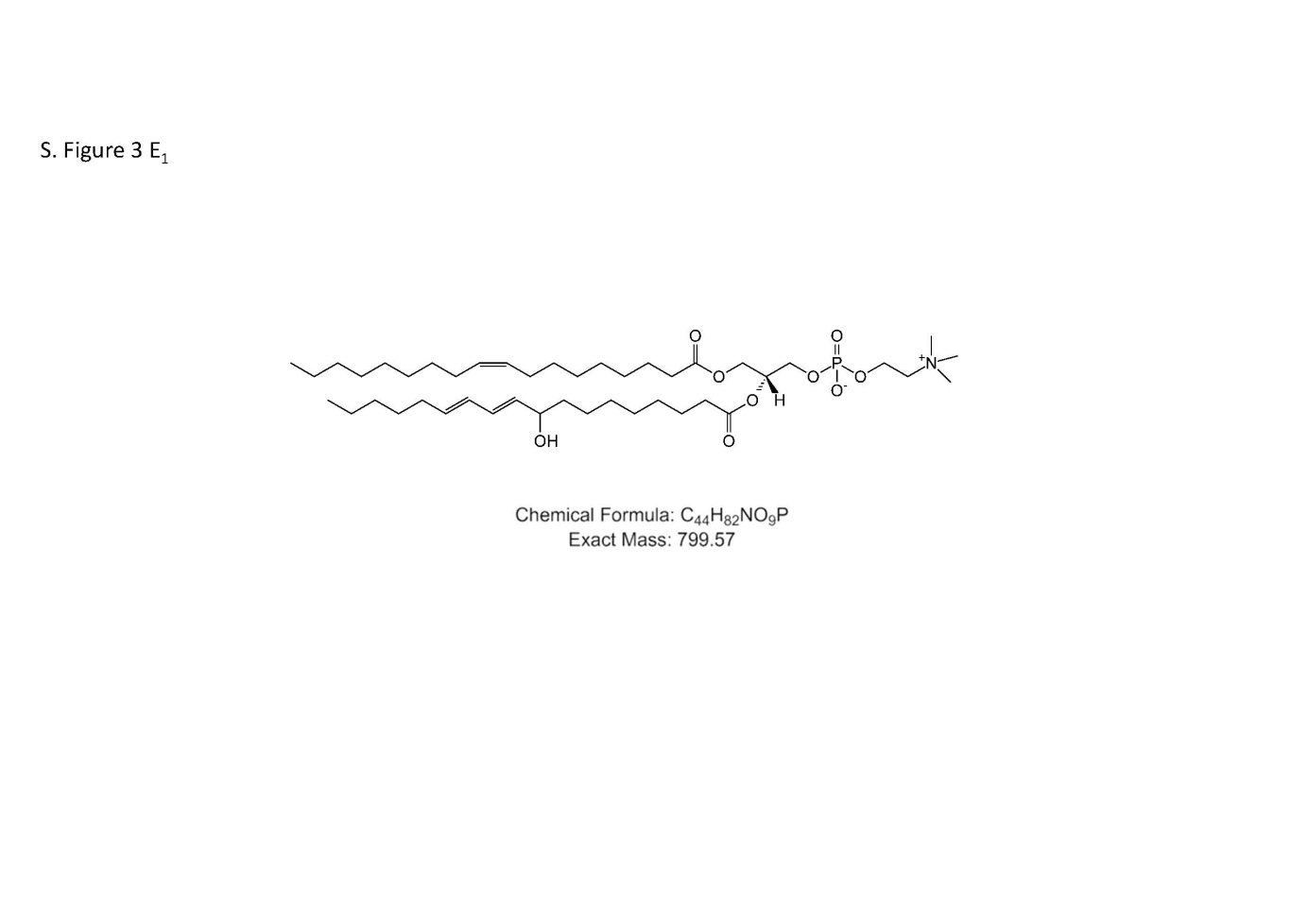
**

**
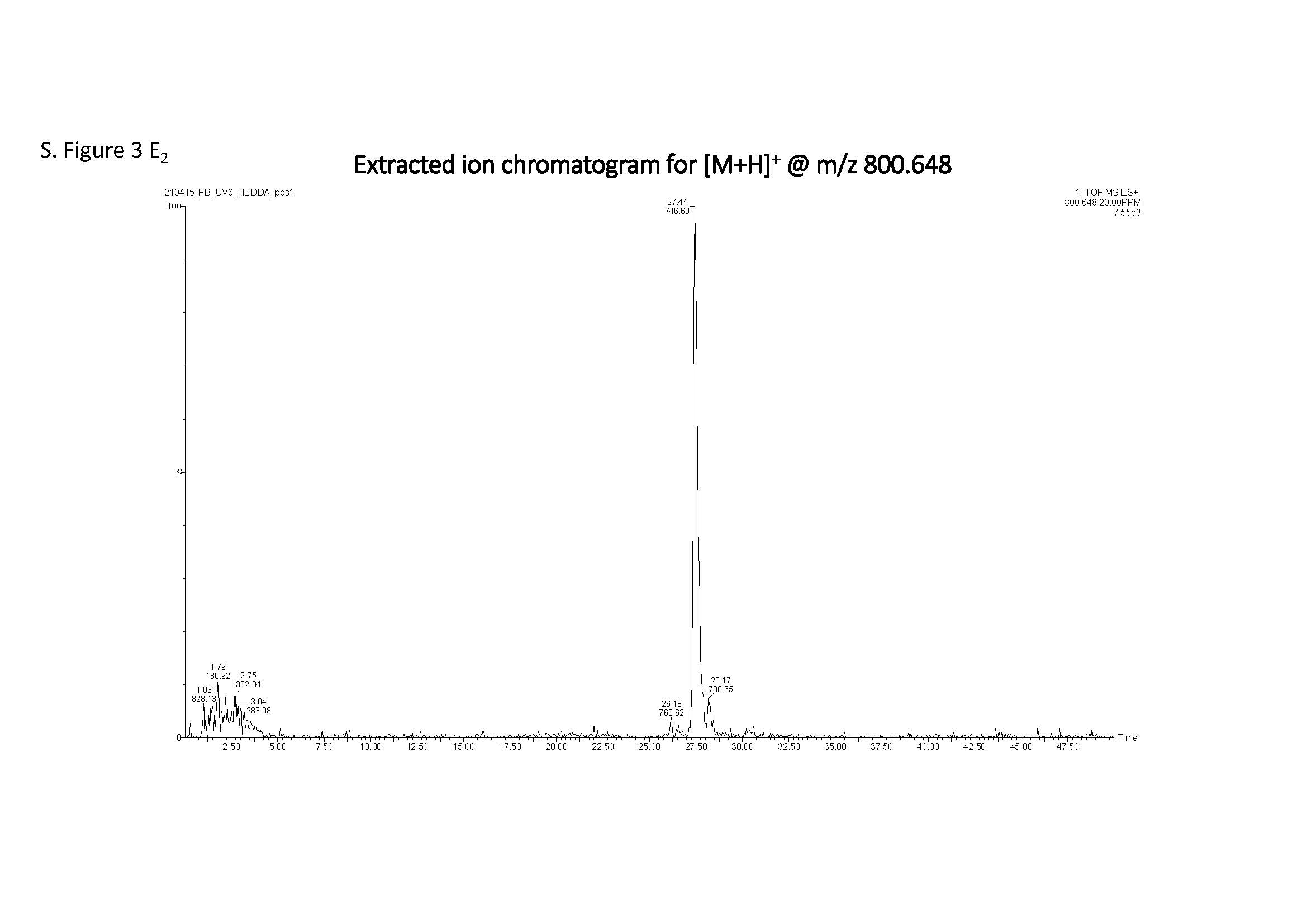
**

**
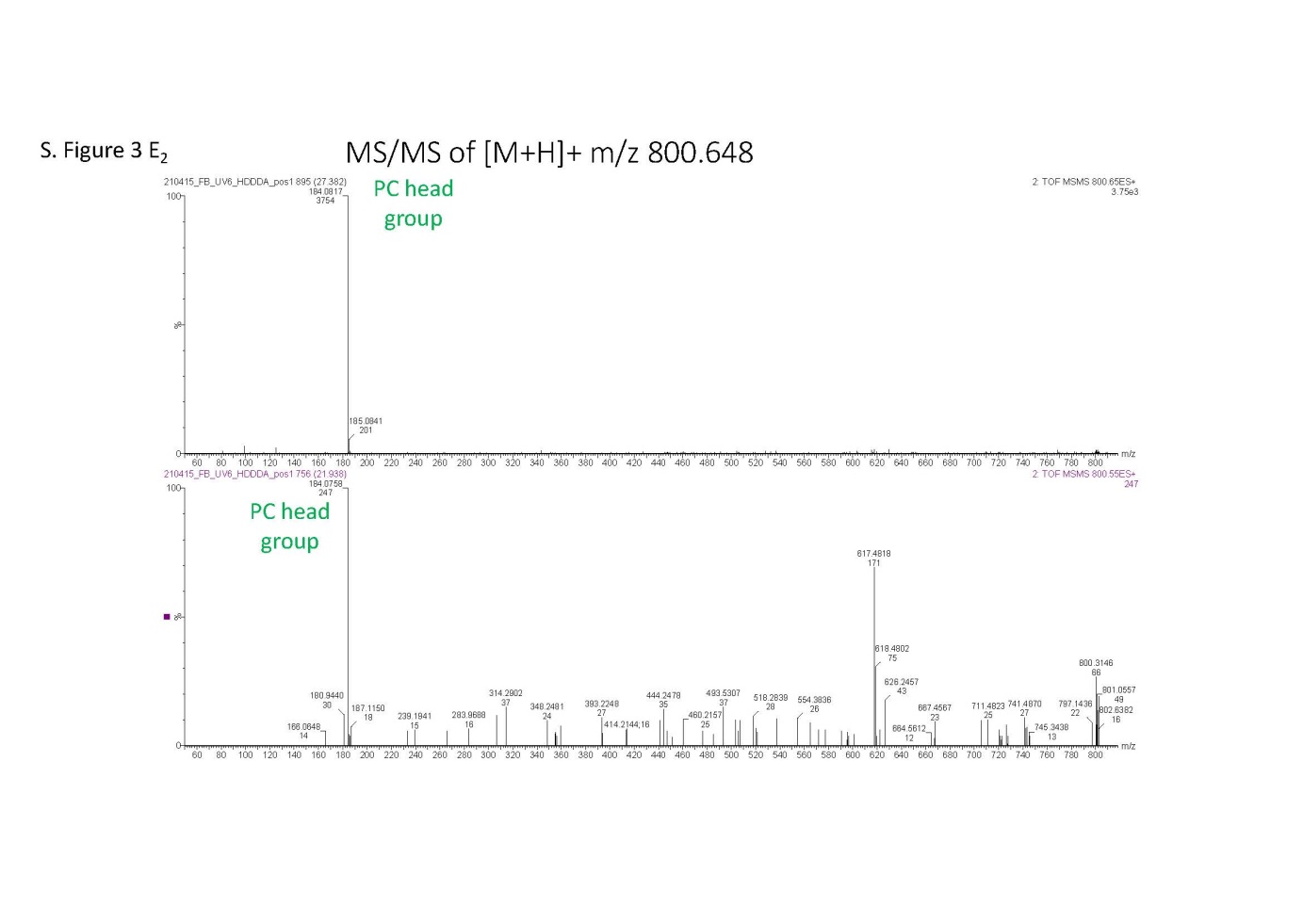

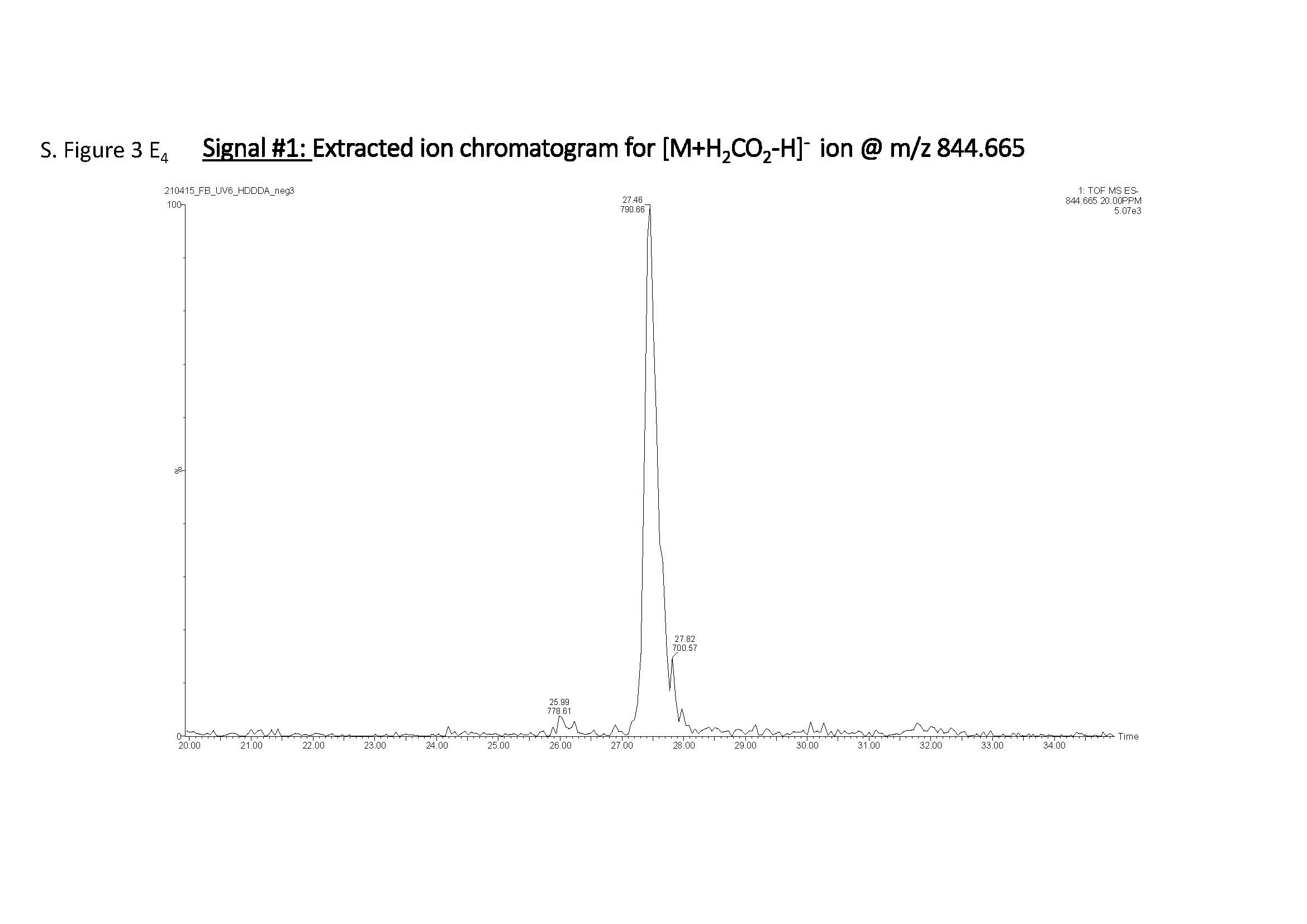

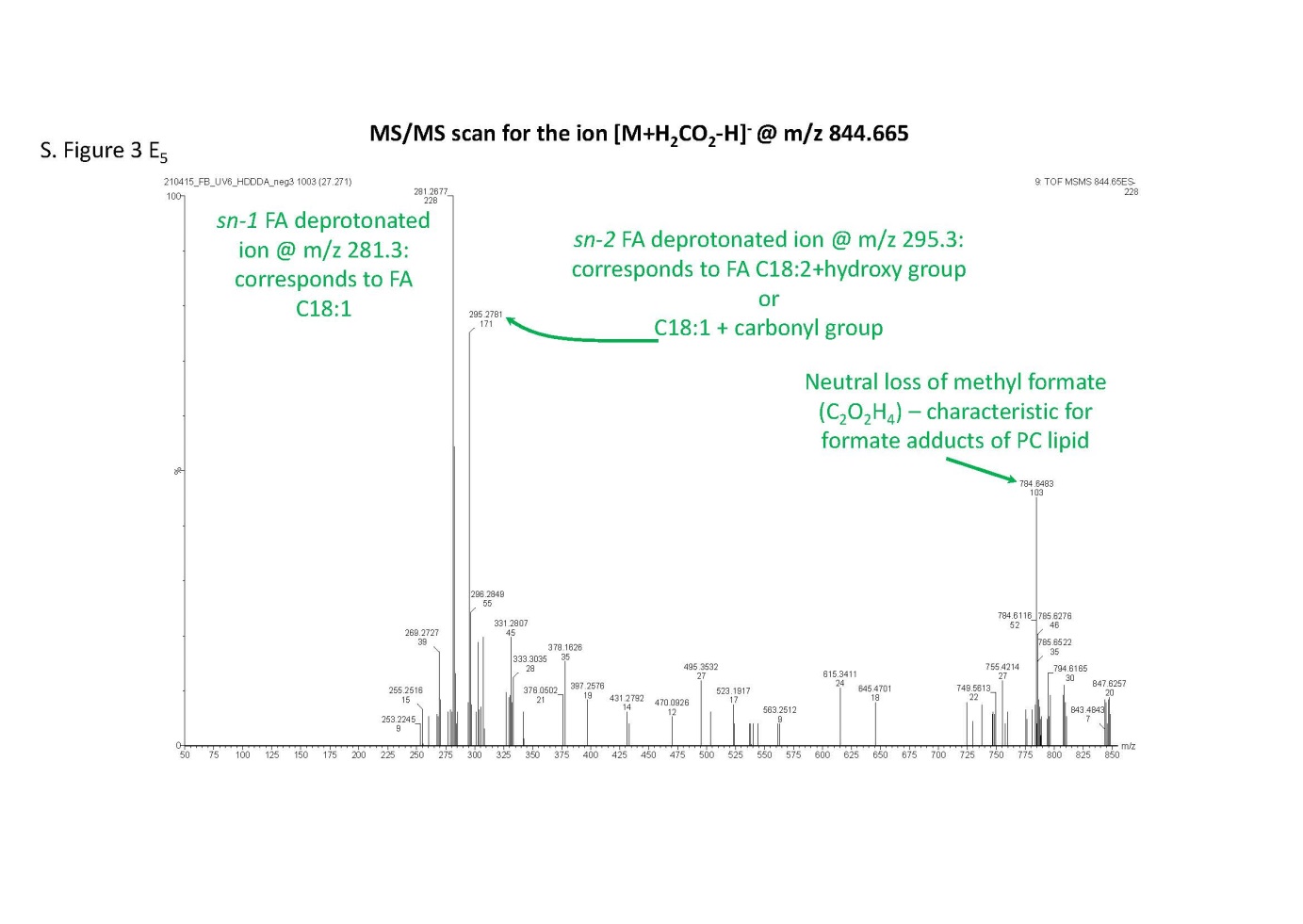
**


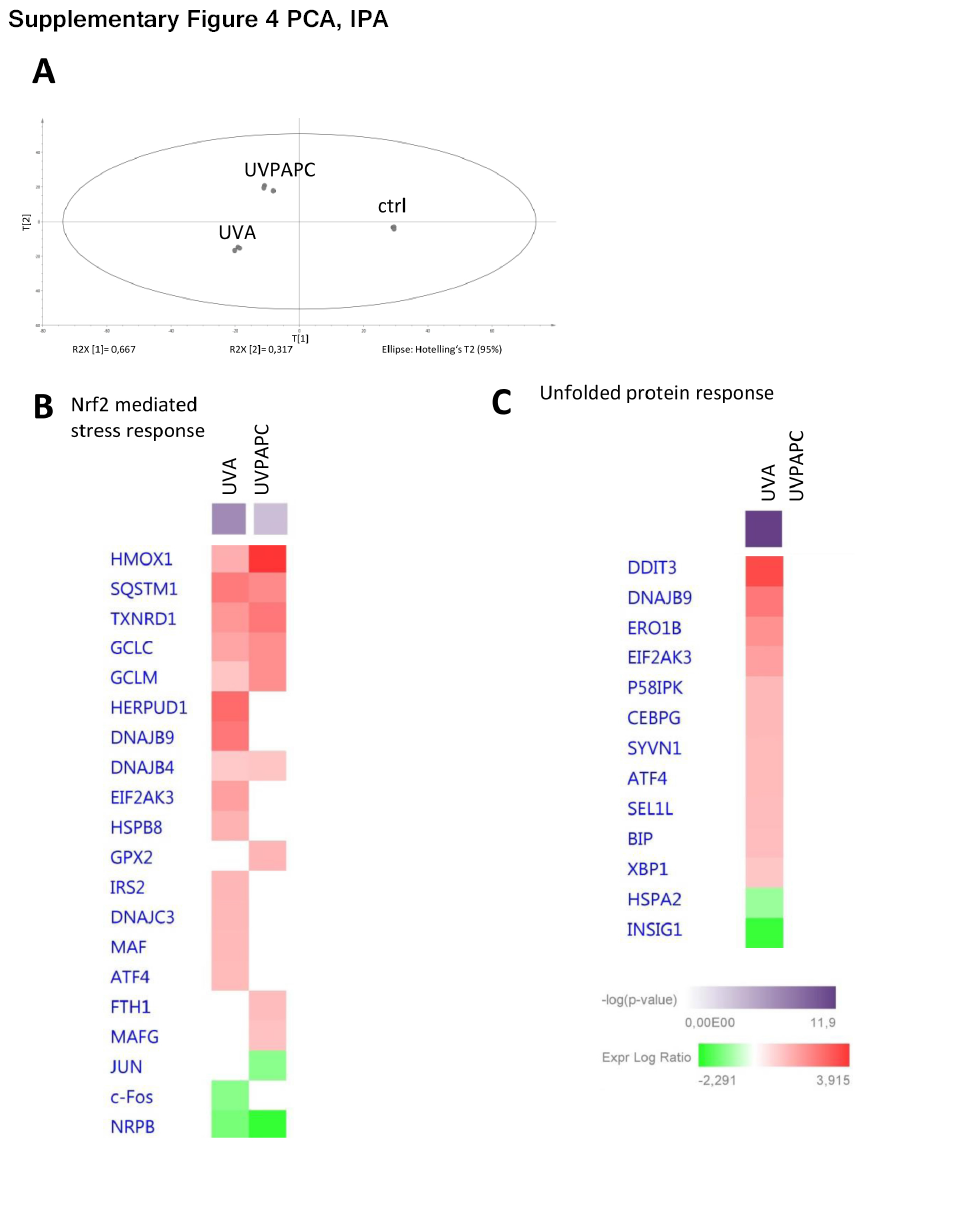


**Supplementary Figure 4. Bioinformatic analysis of the mRNAs of human keratinocytes 7h after UVA and UVPAPC treatment**.

Total RNA was isolated of human primary keratinocytes 7h post stress treatment (UVA-1 40J/cm²; UVPAPC 25µg/ml) and global gene expression was assessed from biological triplicates using microarrays. Principal component analysis (PCA) of mRNA expression data was conducted with all samples (untreated and treated) and is displayed in panel **A.**  Genes that were significantly more than 2 fold regulated and are annotated for unfolded protein response (**B**) and NRF2 mediated stress response (**C**) are listed. Heatmaps show the expression log ratio.

**Supplementary Fig. 5**

**
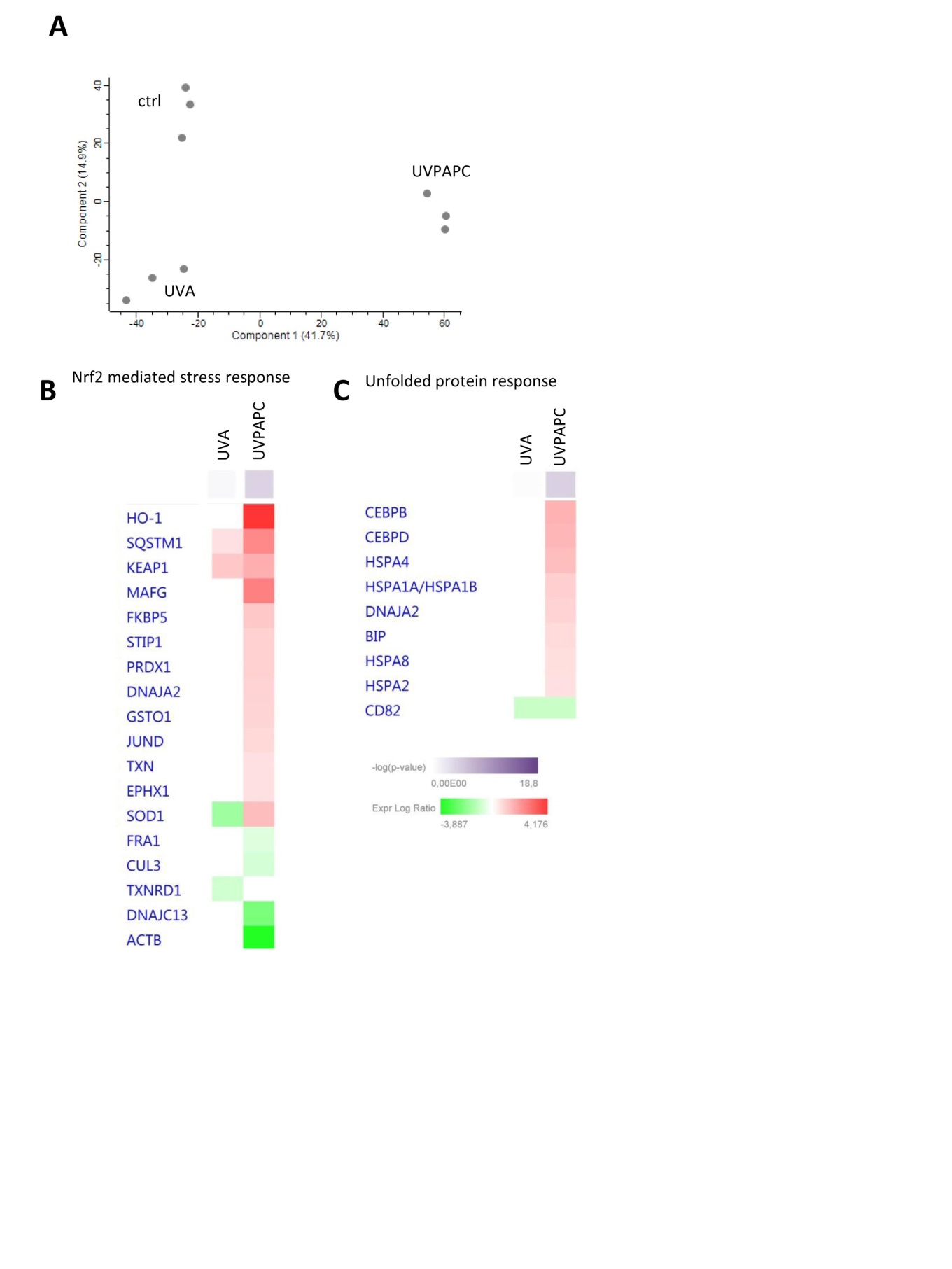
**

**Supplementary Figure 5: Bioinformatic analysis of the protein expression data 24h after UVA and UVPAPC treatment.** Principal component analysis (PCA) of protein expression data was conducted with all samples (untreated, UVA-, UVPAPC- treated) and is displayed in the panel **A.** Genes that were significantly and more than 1.5 fold regulated and are annotated for the UPR (**B**) and NRF2 mediated stress response (**C**) are listed. Heatmaps indicate the expression log ratio.

**Supplementary Fig. 6**

**
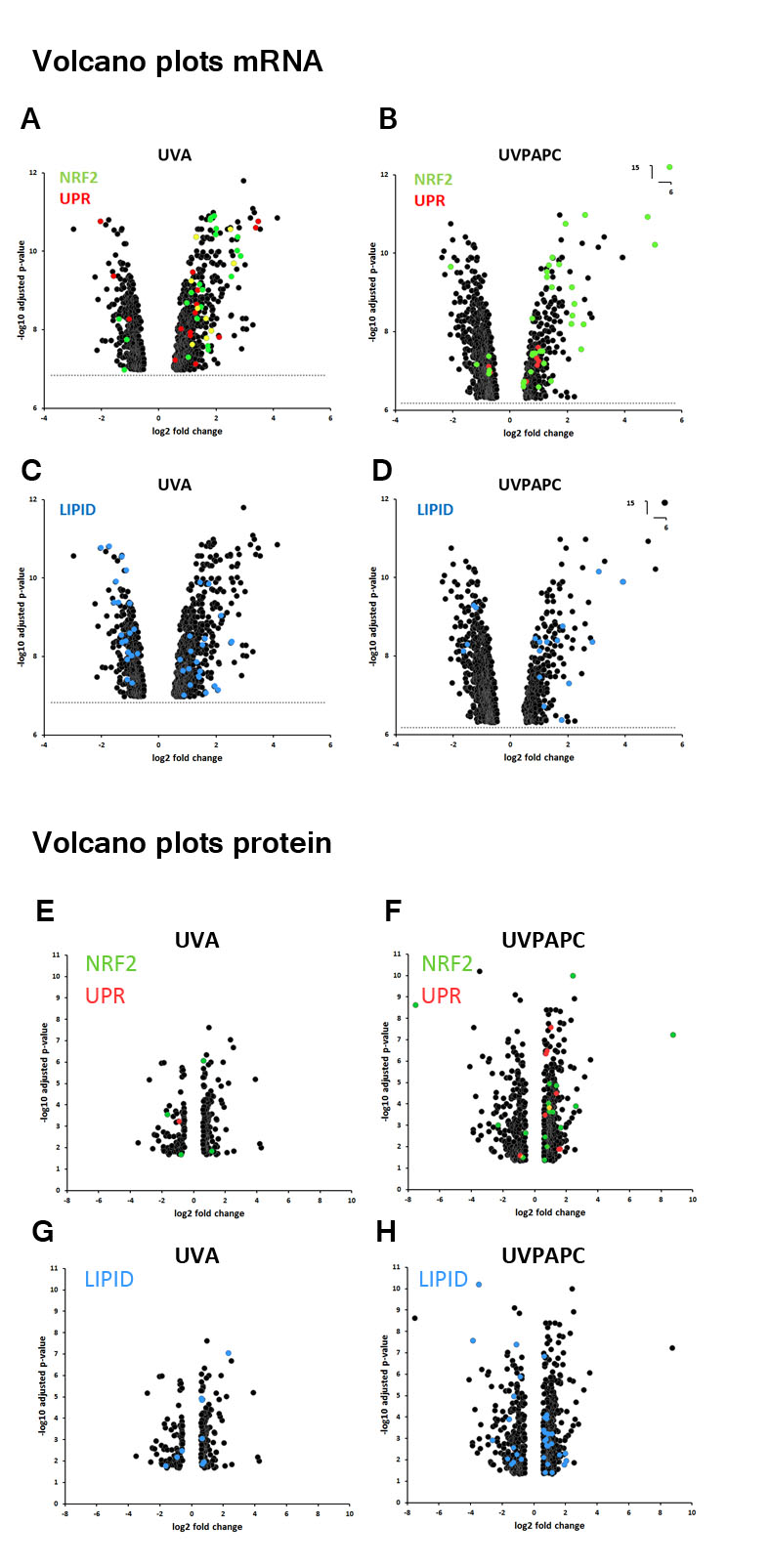
**

**Supplementary Figure 6** - Volcano Plots of regulated mRNAs and proteins.

**A-D** Volcano plots of the top 1000 most significantly regulated transcripts (UVA: A/C; UVPAPC: B/D). Green dots represent the genes annotated for the NRF2 mediated oxidative stress response, red dots genes annotated for the UPR signaling and yellow dots correspond to genes that are annotated for both signaling pathways. Blue dots **(C/D)** represent genes that contain the string “lipid” in their gene ontology entry. The dotted line indicates the top 1000 gene cut off line. **E-H** Volcano plots of the significantly regulated proteins (UVA: **E/G**; UVPAPC: **F/H**). Green dots represent proteins annotated for the NRF2 pathway, red dots represent proteins annotated for UPR signaling and yellow dots correspond to proteins annotated for both signaling pathways. Blue dots represent proteins that contain the string “lipid” in their gene ontology entry **(G/H).**

**Supplementary Fig. 7**


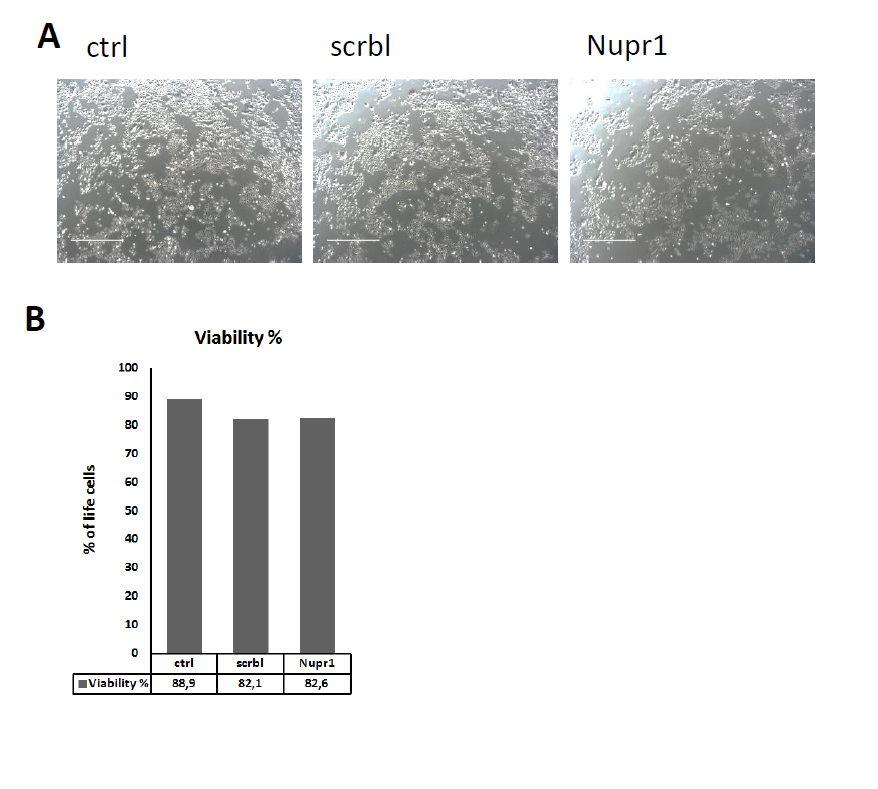


**Supplementary Fig 7. Effects of NUPR1 on keratinocyte cell viability.** Human primary keratinocytes were transiently transfected using a scrambled siRNA or a siRNA targeting NUPR1. **A** Micrographs of cells 48h post transfection. **B** Cell viability was assayed by automated cell counting using an Acridine Orange (AO)/ Propidium Iodide (PI) staining system. Cells positive for AO but negative for PI were counted as live.


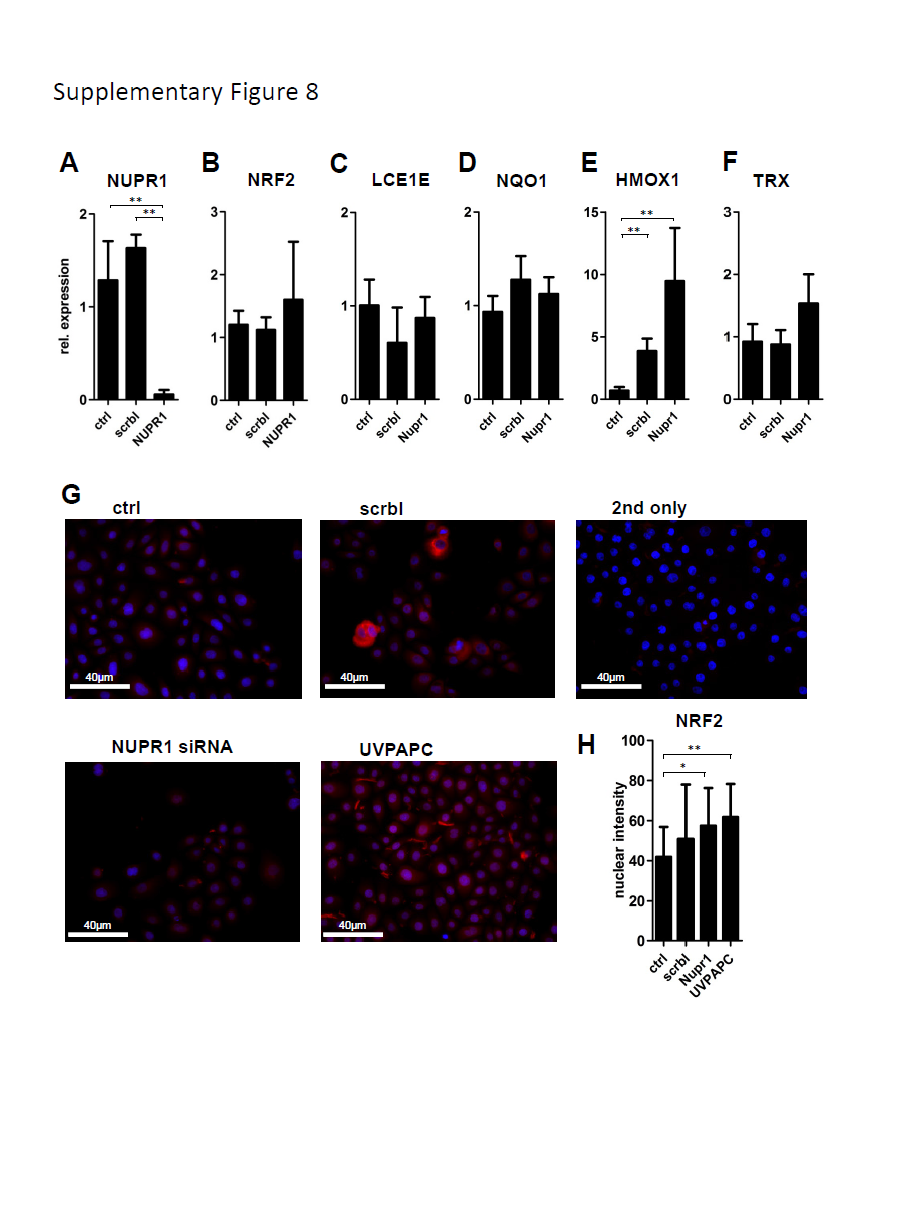
Supplementary Figure 8: **Effects of NUPR1 knockdown on NRF2 activation.**

Human primary keratinocytes were transiently transfected using scrambled siRNA or NUPR1 siRNA. **A-F** Relative mRNA expression of Nupr1 (**A**), NRF2 (**B**), late cornified envelope 1 E (LCE1E, canonical epidermal Nrf2 target gene, differentiation dependent expression) (**C**), canonical NRF2 target gene NQO1 (**D**), HMOX1 (**E**) and TRX (**F**) was quantified by qPCR (n=3; relative quantification normalized to expression of beta-2-microglobulin). Asterisks indicate significant difference (*P<0.05; ** P<0.01) determined by Student’s t-test. **G** NRF2 protein expression in transfected and UVPAPC (25µg/ml) treated primary keratinocytes (immunofluorescence microscopy, NRF2: red, Nuclear stain: blue, representative images). **H** Quantification of nuclear intensity (12 fields of view per condition). Error bars indicate SD; Asterisks show significant differences (*P<0.05; ** P<0.01) determined by Student’s t-test.


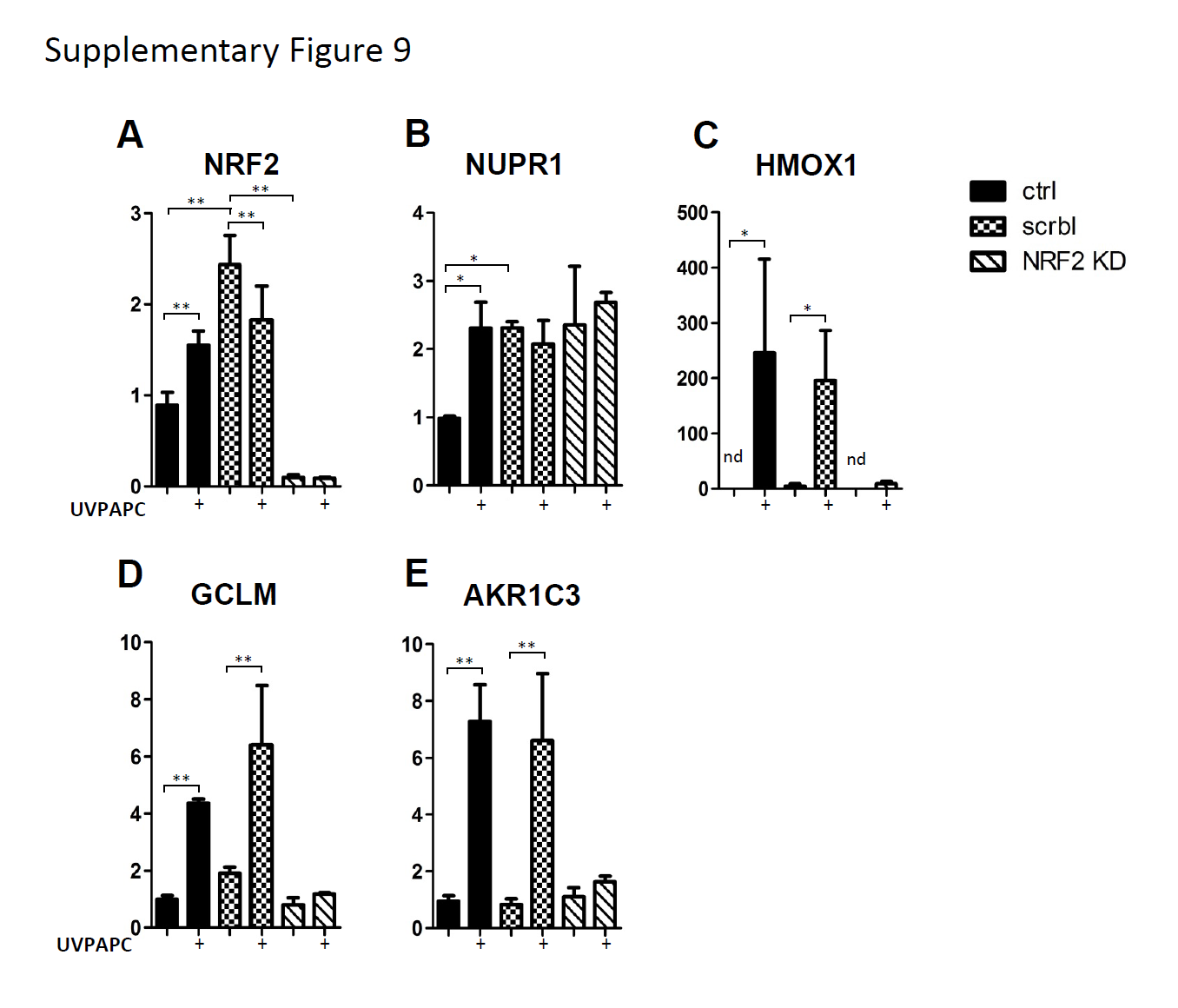


Supplementary Figure 9: **Effect of NRF2 knockdown on NUPR1 expression.**

Human primary keratinocytes were transiently transfected using scrambled siRNA or a siRNA targeting NRF2 and were treated with UVPAPC (25µg/ml). **A-E** 6h post stress treatment relative mRNA expression of NRF2 (**A**), NUPR1 (**B**), HMOX1 (**C**), GCLM (**D**) and AKR1C3 (**E**) was quantified by qPCR (n=3; relative quantification normalized to expression of beta-2-microglobulin). Error bars indicate SD; Asterisks show significant differences (*P<0.05; ** P<0.01) determined by Student’s t-test.

**Supplementary Fig. 10**


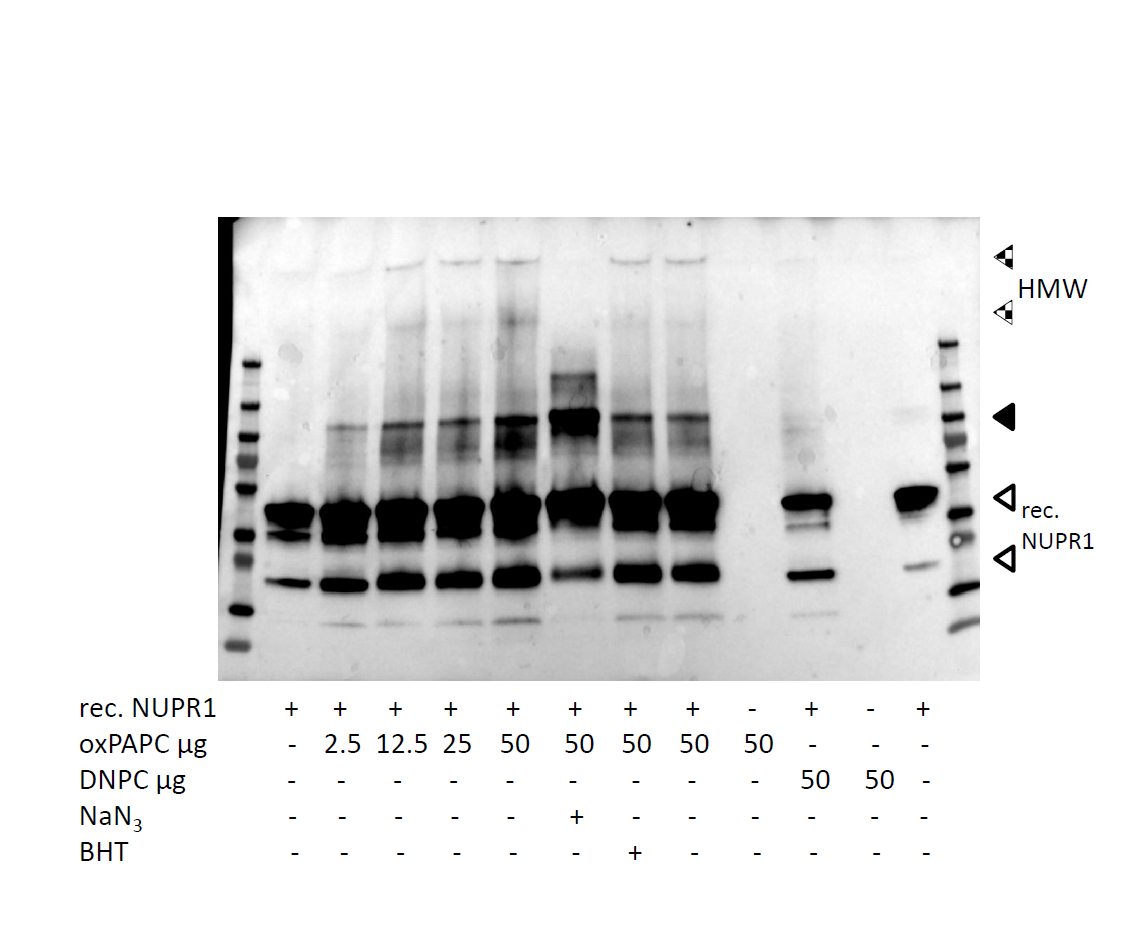


**Supplementary Figure 10 - In vitro oxidation of NUPR1.** Recombinant human NUPR1 protein (150ng) was exposed to oxidized PAPC (2.5 µg to 50 µg) in the presence and absence of sodium azide (NaN_3_, 1mM) or butylated hydroxytoluene (BHT; 0.001%) and to the saturated unoxidized DNPC (50µg). Samples were separated by SDS-PAGE and detected by immunoblotting with an anti-NUPR1 antibody. Checkered arrows indicate high molecular weight crosslinked protein, black arrow indicates a 150 kD band that is inducible by oxidized PAPC treatment and augmented by additional presence of the singlet oxygen quencher NaN_3_. White arrowheads indicate the major and minor bands at which recombinant NUPR1 is detected.
